# Endangered Deep‐Snow Mountain Caribou Have a Distinct Winter Diet and Gut Microbiome That May Be Altered by Maternal Penning

**DOI:** 10.1111/mec.17783

**Published:** 2025-05-07

**Authors:** Scott Sugden, Robert Serrouya, Lalenia Neufeld, Helen Schwantje, Colleen Cassady St. Clair, Lisa Stein, Toby Spribille

**Affiliations:** ^1^ Department of Natural Resource Sciences McGill University Montreal Quebec Canada; ^2^ Department of Biological Sciences University of Alberta Edmonton Alberta Canada; ^3^ Caribou Monitoring Unit Alberta Biodiversity Monitoring Institute Edmonton Alberta Canada; ^4^ Jasper National Park of Canada Parks Canada Jasper Alberta Canada; ^5^ Emeritus, Wildlife Branch, Ministry of Water, Land and Resource Stewardship Government of British Columbia Nanaimo British Columbia Canada

**Keywords:** British Columbia, DNA metabarcoding, gut microbiome, lichen, mountain caribou, wildlife management

## Abstract

Understanding species‐ or population‐specific dietary specialisation is key to informing habitat conservation needs and successful *ex situ* recovery programs for many endangered species. One of the most endangered populations in Canada, the behaviourally distinct deep‐snow ecotype of the Southern Mountain caribou, is characterised by a winter diet of arboreal rather than terrestrial lichens. We hypothesised that this dietary variation would produce a distinct gut microbiome in deep‐snow mountain caribou relative to their shallow‐snow counterparts. We additionally hypothesised that the temporary alteration of natural diets for *ex situ* conservation programs, including the provision of commercial pelleted feed and volunteer‐collected lichens during maternity penning of pregnant cows, may alter this specialised microbiome. Here, we use faecal DNA metabarcoding to compare diet and gut microbiome composition among various herds of deep‐ and shallow‐snow caribou, captive deep‐snow caribou from the Revelstoke maternity pen, and semi‐domesticated reindeer. Our results confirm that free‐ranging deep‐snow caribou specialise on the arboreal hair lichens *Bryoria* and *Nodobryoria,* and we show that this correlates with a microbiome distinct from that of shallow‐snow caribou specialising on the terrestrial lichens *Cladonia* and *Stereocaulon*. We also show that maternity penning of deep‐snow caribou significantly altered forage consumption and microbiome composition: penned caribou consumed more foliose lichens and had a distinct microbiome compared to free‐ranging caribou. Our results suggest that managers should carefully consider the preferred forage of caribou populations when designing interventions that require diet modification. We further suggest that faecal samples of caribou and other dietary specialists be routinely monitored for diet and microbiome composition, especially during periods of captivity or diet modification, as an additional component of conservation assessments.

## 1 Introduction

An increasing number of species worldwide are facing endangerment, extirpation, or extinction due to habitat loss and degradation (Myers et al. 2000). One of the most iconic North American species suffering from these pressures is the caribou ( *Rangifer tarandus* ), which has been extirpated in the contiguous United States but remains as mostly declining populations in Canada, where they are a culturally important resource for Indigenous peoples (Hummel et al. 2008; Sharp and Sharp 2015). Caribou populations are declining across 11 of Canada's 12 “Designatable Units (DU),” conservation units that have been defined for management purposes (COSEWIC 2011). Some of the most significant declines have occurred in what were already some of the smallest populations: the deep‐snow and shallow‐snow mountain caribou in eastern British Columbia and western Alberta (Serrouya et al. 2012), which are respectively classified under the Species at Risk Act into the “Southern group” (DU9) and “Central group” (DU8) from the broader Southern Mountain caribou population (Environment Canada 2014). These two groups of mountain caribou have declined by 64% and 45% in the past few decades, to the point where they now number fewer than 1400 and 500 individuals, respectively (COSEWIC 2014). Although the primary cause of decline in these areas has been attributed to human activities (Mitchell 2021), especially loss of mature forests from clearcut logging (Stevenson 2001), the proximate cause is generally believed to be predation owing to habitat changes that increase predator abundance, access, and mobility (DeMars and Boutin 2018; Dickie et al. 2017; Mumma et al. 2018). As for many North American wildlife populations, the threats facing mountain caribou are further exacerbated by climate change and associated changes in snowpack, fire regimes, forage availability (Greuel et al. 2021; Sullender et al. 2023; Venier et al. 2022), and apparent competitors (Dickie et al. 2024; Holt 1977). Given these ongoing pressures, the conservation of all mountain caribou is increasingly urgent, and recovery actions must include all tools available.

The various drivers of caribou population declines are exacerbated by their high degree of dietary specialisation, which is a frequent contributor to species endangerment in diverse species (Purvis et al. 2000) and can pose challenges to captivity‐based or *ex situ* conservation efforts (Slatyer et al. 2013). These challenges are magnified by seasonal dietary variation between deep‐ and shallow‐snow caribou, which is associated with the distinct habitats and evolved migratory behaviours of the two caribou ecotypes (Webber et al. 2022). In the summer, both caribou ecotypes forage on vascular plants native to their respective habitats, but in the late winter, deep‐snow mountain caribou west of the continental divide migrate to high‐elevation, old‐growth forests. The deep snowpacks (up to 3–4 m) in these habitats limit both the growth and accessibility of terrestrial forage typical of other Canadian caribou; instead, deep‐snow caribou stand atop the snowpack to reach their primary winter food source of arboreal lichens in mature conifers, preferring hair lichens of the genus *Bryoria* over other arboreal taxa (Antifeau 1987; Edwards and Ritcey 1960; Edwards et al. 1960). Some deep‐snow subpopulations perform a biannual altitudinal migration, a behaviour that may be globally unique among ungulates (Apps et al. 2001). After spending the winter in high‐elevation habitats, these caribou move downslope to snow‐free areas in the spring, where they continue to consume lichens but also forage on fresh plant growth. Throughout the late spring and summer, they slowly migrate back upslope to follow the “green‐up line,” returning to low‐elevation habitats to maintain their primarily plant‐based forage in the fall before climbing again to their winter forage of arboreal lichens (Seip 1990). In contrast, shallow‐snow caribou east of the continental divide spend the winter in coniferous forests or on windswept alpine slopes, where they paw through the snow for terrestrial lichens such as *Cladonia* and *Stereocaulon* as well as small amounts of plant material (Cichowski 1993; Denryter et al. 2017).

Studies of diverse animal taxa have emphasised the close relationship between an animal's diet and the composition of its gut microbiome, especially among dietary specialists for which obtaining adequate nutrition often requires an equally specialised gut microbial community (de Jonge et al. 2022; Liu et al. 2021; Youngblut et al. 2019). However, the gut microbiome has not been assessed previously in mountain caribou, with only one previous study of woodland caribou populations in central Canada (Nagati et al. 2024). In other ruminants, gut microorganisms digest the complex polysaccharides found in plant‐ or lichen‐based diets, producing short‐chain fatty acids that provide a readily available energy source (Mizrahi et al. 2021; Moraïs and Mizrahi 2019). Because different microbial taxa digest different substrates, ruminant microbiome composition is closely associated with the type, quality, and fermentability of fibre in the animal's diet (Glendinning et al. 2021; Parnian‐Khajehdizaj et al. 2023; Terry et al. 2019), although host sex, age, and species identity can also structure the gut microbiota (Härer and Rennison 2023; Macke et al. 2017). For caribou, arboreal and terrestrial lichens are comprised of different structural polysaccharides—β‐linked glucans in arboreal lichens like *Bryoria* vs. α‐linked glucans in terrestrial lichens like *Cladonia* (Common 1991; Olafsdottir and Ingólfsdottir 2001; Spribille et al. 2020; Svihus and Holand 2000)—that are degraded by different microbial metabolic pathways (Suyotha et al. 2016). These subtle chemical differences, combined with the narrower range of winter forage consumed by deep‐snow caribou, would be expected to result in winter microbiome differentiation between deep‐ and shallow‐snow caribou. Characterising the caribou microbiome could therefore further reveal the nature of winter variation among these populations, providing additional ecological data to inform conservation planning, monitoring, and management actions.

One such management action used to increase small caribou populations, but with unexplored effects on their gut microbiome, is an *ex‐situ*, temporarily‐captive management action termed maternity penning. In these programmes, mature, pregnant female caribou are captured prior to calving and held in captive pens in native habitats during the calving period, protected by fencing and guards to reduce predation on neonatal calves (Adams et al. 2019; Serrouya et al. 2019; Smith and Pittaway 2011). Due to the inability to provide a 100% natural diet from the pen itself, temporary captivity in maternal pens requires that free‐ranging caribou are transitioned away from their lichen diet to one consisting of a commercial pelleted feed designed for caribou (Serrouya, Bollefer, et al. 2021), and this transition could alter their natural microbiome composition. This potential alteration is important because the lack of a robust native microbial community in penned animals may compromise animal health and reintroduction success (Trevelline et al. 2019; West et al. 2019). For caribou, current maternity penning protocols include a transition period on arrival in the pen from free‐ranging natural diets to a hand‐picked lichen diet, to a diet of 100% pelleted feed at a rate of 10% addition of pellets per day over at least 2 weeks. A second transition period back to a hand‐picked lichen diet occurs prior to release. Cow‐calf pairs are usually released from the pens by mid‐summer after calves are at least 1 month of age (Legebokow 2016).

To date, three maternity pen programs have been established in British Columbia. The ongoing Klinse‐Za maternity pen project for shallow‐snow mountain caribou was accompanied by significant wolf control efforts in the area of the herd home range and has reduced calf mortality and increased effective population size (Lamb et al. 2022; McNay et al. 2022). Among deep‐snow caribou, the Central Selkirk/Arrow Lakes program began in 2022 for the Central Selkirks subpopulation, but little analysis has been performed to date (Arrow Lakes Caribou Society 2023). The Revelstoke Caribou Rearing in the Wild program (2013–2018) for the Columbia North subpopulation had years with high mortalities of adult females as well as calves, as compared to other penning programs; post‐program data analysis suggested that the caribou were subjected to warm temperatures that exceeded their thermal limits due to a combination of pen location, altitude, and weather patterns, among other factors (Serrouya, Bollefer, et al. 2021). All these penning projects included natural habitats of varying sizes though none had the information needed to match specific lichens that were hand‐picked or available in the pens to population‐specific diets and microbiomes.

Given this ecological context, we hypothesised that (i) the winter diet of arboreal lichen for deep‐snow mountain caribou requires a microbiome distinct to that of shallow‐snow mountain caribou and (ii) diet alteration in the course of maternity penning and supplemental feeding would alter the caribou's natural microbiome. The latter hypothesis stems from several studies showing that the microbiome of captive and/or pellet‐fed animals differs from that of wild and/or forage‐fed conspecifics (Alberdi et al. 2021; McKenzie et al. 2017). For example, captive European reindeer fed pelleted feed had an altered microbiome relative to reindeer that were fed lichens (Salgado‐Flores et al. 2016) and their rumen extract was less efficient at digesting lichens (Storeheier et al. 2002). Furthermore, the effect of supplemental or artificial feeding is likely to be greater in dietary specialists maintained in captivity because they lose a greater proportion of their native gut microbiota than dietary generalists (Kohl et al. 2014). Because *ex situ* management is a recognised method of recovery of endangered species and has gained support for caribou recovery efforts, exploring the mountain caribou microbiome could contribute essential information for future conservation actions. Specifically, such information could provide a tool for monitoring dietary adaptation both in animals receiving supplementary feed in situ, such as those in the Kennedy Siding subpopulation of shallow‐snow caribou (Heard and Zimmerman 2021) and in animals moving in and out of captivity, including ongoing and future *ex situ* recovery programmes in Klinse‐Za, the Central Selkirks (Arrow Lakes Caribou Society 2023), Jasper National Park (Parks Canada Agency 2022), and elsewhere. This information applies not only to caribou but to endangered species worldwide for which conservation action requires short‐ or long‐term diet modification.

We therefore aimed to test the effect of winter dietary specialisation and *ex situ* management on the gut microbiome of different mountain caribou and *Rangifer* ecotypes. Although we suspect that the caribou gut microbiome may vary seasonally with their diet, we focused our sampling on winter because this is when among‐population differences in diet and habitat use are most pronounced and pregnant females are moved into maternity pens. Our specific objectives were to (a) characterise the winter lichen diets of multiple free‐ranging subpopulations of deep‐ and shallow‐snow caribou to verify previous distinctions between these two types, (b) determine whether the highly specialised winter diet of deep‐snow caribou is reflected in a similarly distinct microbiome, and (c) measure changes in the microbiome associated with maternal penning in at least one penning project. We did so by using DNA metabarcoding of faecal samples to compare both diet and microbiome composition for free‐ranging and penned caribou. As a reference for the effects of pellet diets, we compared our data to the microbiome of a semi‐domesticated, captive reindeer population that rarely receives lichens. Our sequencing efforts targeted fungal and plant ITS2 gene amplicons, bacterial/archaeal 16S rRNA gene amplicons, and eukaryotic 18S rRNA gene amplicons to detect signatures of natural diet (lichens, algae, and plants) and gut microbiome composition (bacteria and protists). Based on our hypothesis that population‐specific diets require correspondingly specialised microbiomes, we predicted that deep‐snow caribou consuming a select few genera of arboreal lichens would have a distinct and less species‐rich gut microbiome relative to shallow‐snow caribou consuming a range of terrestrial lichens and plant material. Based on our hypothesis that captivity and pelleted feed reshapes the wild‐type microbiome of dietary specialists like caribou, we predicted that caribou provided with pellets would have an altered microbiome with reduced relative abundances of the major carbohydrate‐degrading taxa present in free‐ranging populations.

## 2 Materials and Methods

### 2.1 Sample Collection

**FIGURE 1:**
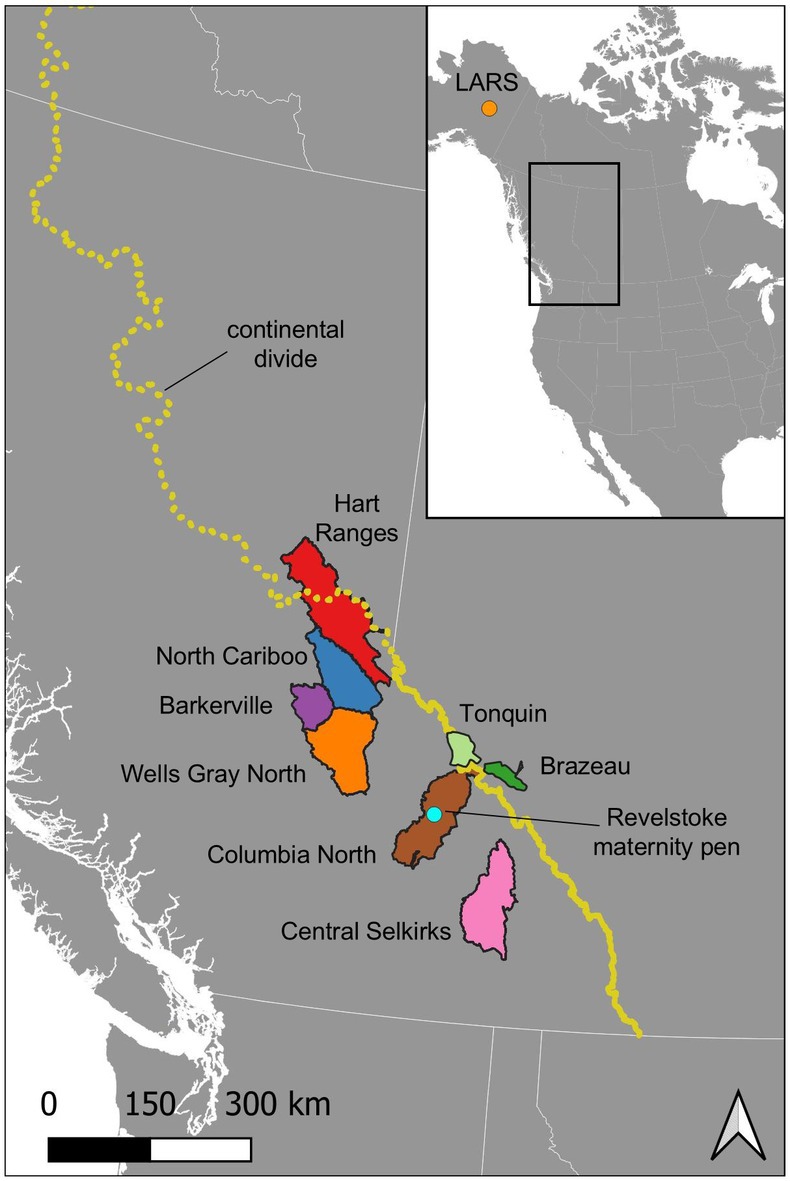
Map of the study area and caribou subpopulation boundaries. Map showing caribou subpopulation boundaries in eastern British Columbia and western Alberta. Home ranges of the deep‐snow mountain caribou subpopulations sampled as part of this study are indicated in different colours, and home ranges of the Tonquin and Brazeau subpopulations of shallow‐snow caribou are shown in shades of green. The yellow dotted line indicates the continental divide, which largely separates deep‐snow and shallow‐snow caribou habitats. The locations of the Revelstoke maternity pen and the Large Animal Research Station (LARS) are indicated with turquoise and orange dots, respectively.

We obtained faecal samples from two free‐ranging, one temporarily captive, and one permanently captive group of caribou, encompassing the two endangered mountain ecotypes as well as individuals receiving pelleted feed. For the deep‐snow mountain caribou, we obtained samples from six free‐ranging subpopulations: the Barkerville, Wells Gray North, North Cariboo, Central Selkirks, Hart Ranges, and Columbia North herds, all on the west side of the continental divide (Figure 1). Deep‐snow faecal samples were collected in January–February 2021 and were provided to us by the British Columbia Wildlife Health Program. For the shallow‐snow caribou on the east side of the continental divide, we obtained samples from the Brazeau and Tonquin subpopulations in Jasper National Park (Figure 1). Shallow‐snow mountain caribou faecal samples were collected in November 2020 and provided to us by Parks Canada. For all free‐ranging caribou, samples were collected within no more than 24 h of defecation during routine surveys and collaring projects.

To test the effects of captivity and pelleted feed on the gut microbiome, we also obtained faecal samples from one maternity‐penned group of caribou and one semi‐domesticated reindeer population. First, samples were collected from deep‐snow mountain caribou held in the Revelstoke Caribou Rearing in the Wild Maternity Pen (hereafter “Revelstoke Pen”), a 9.3‐ha fenced area located at 580 m elevation in a sparsely restocked clearcut near Lake Revelstoke (Figure 1; Serrouya, Bollefer, et al. 2021). Faecal samples were collected in 2017, the fourth season (of five) that the maternity pen was in operation. All samples were collected in May; this collection period was later than for free‐ranging caribou but was necessary to ensure that caribou in the pen had been fully transitioned to a diet of pelleted feed. Caribou were provided with 3.2 kg pellets per animal per day (Serrouya, Bollefer, et al. 2021). Limited naturally occurring lichen forage was present within the pen, but this forage had been largely depleted during the initial years of the penning operation (R. Serrouya, personal observation). We additionally obtained samples from reindeer held in year‐round captivity at the Large Animal Research Station (LARS) in Alaska. The reindeer held in this 54‐ha enclosure are sustained year‐round on a diet of pelleted feed, with lichens only provided as an occasional treat (J. Blake, personal communication). All samples were collected within 1–3 h of defecation.

### 2.2 Multiplex Amplicon Sequencing

All faecal samples were stored and transported at −20°C and remained frozen until DNA extraction. For DNA extraction, samples were manually homogenised using a mortar and pestle, and 100 mg of faecal material was processed using the Qiagen DNeasy PowerSoil Pro DNA extraction kit. We screened the DNA samples for purity and yield and sent the extracted DNA to Genome Quebec (Montreal, QC) for PCR amplification, library preparation, and sequencing. In brief, each DNA sample was PCR‐amplified in four separate reactions, with one reaction for each of four different primer pairs: 515F/926R to target the 16S rRNA gene, 565F/948R to target the 18S rRNA gene, ITS9F/ITS4R to target the fungal ITS2 region, and ITS2F/ITS3R to target the plant ITS2 region (see Table S1). We chose these primer pairs because they provide information on both caribou diet (lichens and plants) and gut microbiome composition (bacteria and eukaryotes) (Nagati et al. 2024). The forward and reverse PCR primers were modified to include Illumina sequencing adapters and indexes such that amplification and dual indexing occurred in the same PCR reaction. Following amplification, samples were pooled at equimolar concentrations and sequenced on an Illumina MiSeq using v3 chemistry and 300 bp paired‐end reads. Samples were sequenced alongside negative controls and a mock community of known composition (ZymoBiomics Microbial Community DNA Standard).

Amplicon sequences were quality‐filtered and error‐corrected using the DADA2 algorithm implemented in R 4.2.3 (R Core Team 2019) and then clustered into operational taxonomic units (OTUs) at 97% sequence identity. We removed OTUs with an overall relative abundance of less than 0.001%, OTUs that were not the expected length for each amplicon and samples that produced fewer than 5000 quality‐filtered reads. Bacterial, eukaryotic, and fungal OTUs were given taxonomic assignments based on the RDP v18 (Cole et al. 2014), SILVA v138 (Quast et al. 2013), and UNITE v9.0 (Abarenkov et al. 2022) databases, respectively, and plant OTUs were given taxonomic assignments based on the results of a nucleotide BLAST search. Refer to the Data S1 for a detailed description of our sequence processing workflow. All four amplicon feature tables were imported into the R package *phyloseq* (McMurdie and Holmes 2013) for downstream analysis.

### 2.3 Statistical Analysis

To isolate diet‐based signatures from our metabarcoding data, we created additional subsets of our data that included (i) only fungi known to form lichen symbioses (hereafter “lichen fungal symbionts”) and (ii) only algae, which are another important component of lichen symbioses. For this study, we identified lichen fungal symbionts as any fungal genera defined as such by either Lücking et al. (2016) or FUNGuild (Nguyen et al. 2016). We then manually reviewed the list of remaining taxa to remove those erroneously classified as lichens (see Data S1). Algal OTUs were identified based on their taxonomic classification in the SILVA database. By creating separate feature tables for lichen fungal symbionts and algae, we ensured that the relative abundances of individual diet items (e.g., lichen fungal genera) were presented as a proportion of all the diet items (e.g., all lichens) that we detected. In other words, estimates of diet composition were not skewed by the presence of any non‐diet items (e.g., coprophilic fungi) that were also detected using our PCR primers. Lastly, we created a third subset of our data that separated protists from other 18S rRNA gene amplicons, which allowed us to evaluate the eukaryotic members of the gut microbiome separately from any fungal and plant sequences that were better captured by the two pairs of ITS primers. We did not assess the fungal component of the microbiome due to the difficulty in distinguishing environmental fungi or foraging bycatch from true gut residents.

For each sample and amplicon or amplicon subset (lichen fungal symbionts, algae, and protists), we calculated total OTU richness and Shannon diversity from raw read counts using *iNext*, which produces asymptotic estimates for these values based on rarefaction curves and thereby mitigates confounding effects of read depth (Hsieh et al. 2016). We then used ANOVAs followed by Tukey's post hoc test to screen for significant differences in alpha diversity among study groups and subpopulations. We visualised beta diversity among study groups and subpopulations using principal coordinate analysis based on the Aitchison distance, which involves a centered log‐ratio transform of the raw sequence counts to account for the compositional nature of sequencing data. Significant differences in community composition were then identified using permutational multivariate analyses of variance (PERMANOVAs) and tests for homogeneity of multivariate dispersion. Differentially abundant taxa among study groups and subpopulations were identified using *ALDEx2* (Fernandes et al. 2013) and random forest models (Liaw and Wiener 2002). Wherever appropriate, *p*‐values were adjusted for multiple comparisons using the false discovery rate correction, and significance was defined at *p* < 0.05.

We then tested whether gut microbiome composition could be explained by diet composition and, if so, which microbial groups were most closely associated with specific diet items. First, we used redundancy analysis (RDA) to assess the extent to which overall microbiome composition could be explained by overall dietary composition, with separate RDA models for each pairwise comparison between microbiome (bacteria or protists) and diet (lichens or algae) data sets (see Data S1). Second, we tested for co‐occurrence patterns between the relative abundances of specific members of the gut microbiota (bacteria or protists) and the relative abundances of specific diet items (lichens or algae) using Spearman correlations. For each amplicon or amplicon set, the relative abundances of each genus were correlated with the relative abundances of (i) every other genus in the same amplicon set and (ii) the relative abundances of every genus in every other amplicon set. We corrected *p*‐values for multiple comparisons, as before, and then retained the 179 correlations for which Spearman's *r* > 0.6 and FDR‐adjusted *p* < 0.01. Network statistics, including the betweenness centrality, closeness centrality, and degree for each taxon in the network, were then calculated using CytoScape v3.10.0 (Shannon et al. 2003). Hub taxa were defined as the most interactive genera in the network as a function of their degree, betweenness centrality, and closeness centrality (see Data S1).

## 3 Results

Overall, we collected a total of 67 caribou faecal samples across our four study groups, and our sequencing effort produced 7,305,679 high‐quality reads distributed across our four target amplicons (Table 1). Twenty‐four samples from deep‐snow caribou failed to produce any reads for the plant ITS2 amplicons, but otherwise, only one deep‐snow caribou sample was lost due to low sequencing depth in both the 16S and 18S amplicons (Table 1). Sequencing controls performed as expected (see Figures S1 and S2).

**TABLE 1:** Sample overview.

Ecoregion & herd	Total N	Number of reads (number of samples)							
		16S		18S		Fungal ITS2		Plant ITS2	
*Southern Mountain*	**29**								
Barkerville	5	79,308	(5)	103,633	(5)	176,277	(5)	31,345	(1)
Central Selkirks	3	31,226	(3)	59,840	(3)	116,478	(3)	72,681	(3)
Hart Ranges	7	119,023	(7)	119,906	(7)	223,142	(7)	30,257	(1)
North Cariboo	4	45,478	(4)	80,218	(4)	117,792	(4)	0	(0)
Columbia North	5	57,216	(4)	90,258	(4)	197,148	(5)	0	(0)
Wells Gray North	5	69,932	(5)	98,389	(5)	158,951	(5)	0	(0)
*Central Mountain*	**10**								
Brazeau	3	143,575	(3)	98,459	(3)	113,199	(3)	95,158	(3)
Tonquin	7	293,478	(7)	211,785	(7)	200,531	(7)	190,379	(7)
Revelstoke Pen	**15**	630,707	(15)	399,437	(15)	506,730	(15)	377,867	(15)
Large Animal Research Station	**13**	570,698	(13)	465,988	(13)	470,644	(13)	458,742	(13)

*Note:* Table shows the number of faecal samples collected from each population or herd (“Total N”) as well as the number of high‐quality sequencing reads produced for each amplicon. Because some samples failed to amplify or produce enough reads for analysis, numbers in parentheses indicate the number of individuals that were used for analysis for each amplicon.

### 3.1 Evidence of Unique Diets

#### 3.1.1 Lichen Fungal Symbionts

**FIGURE 2:**
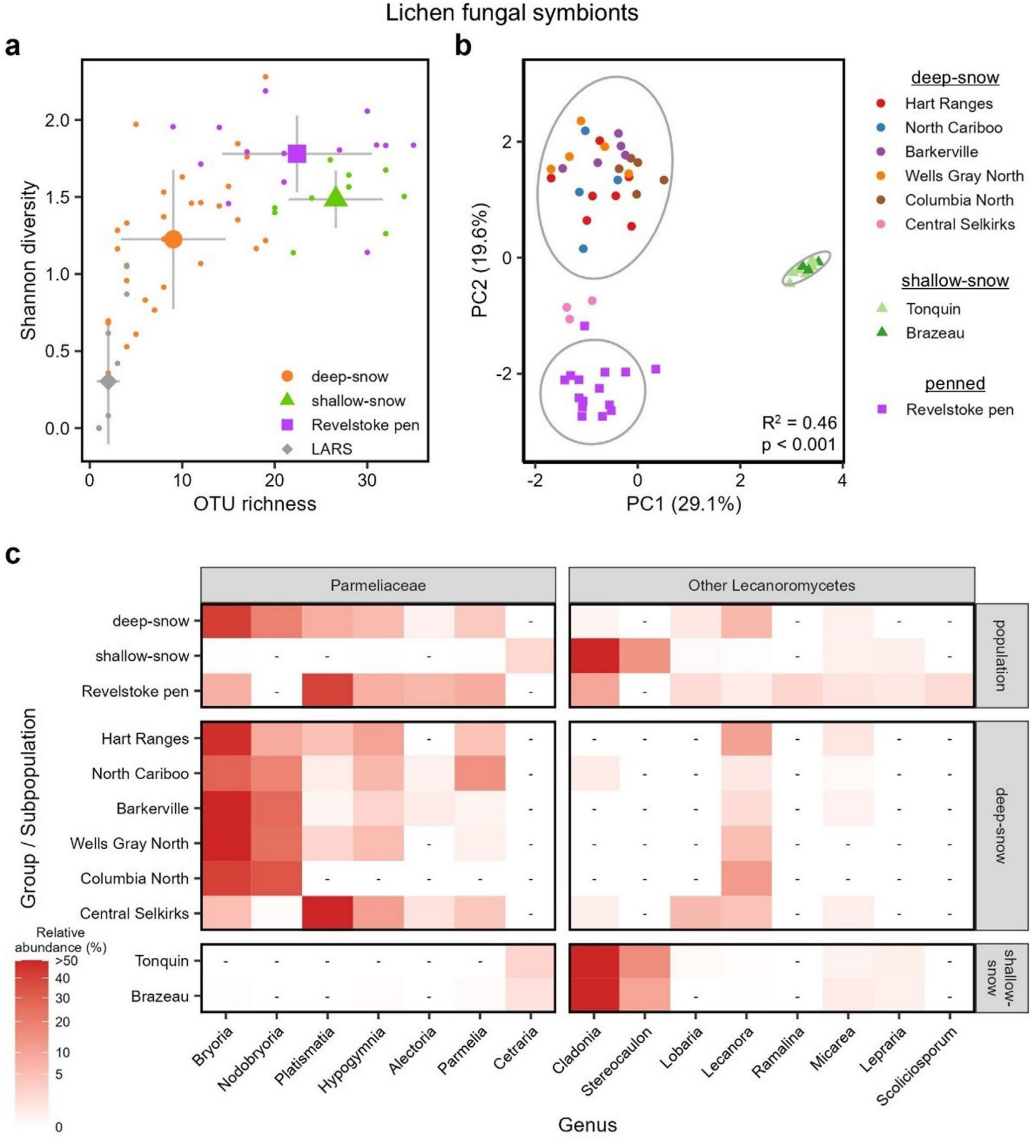
Differences in lichen fungal symbiont diversity and composition among study groups. (a) Lichen fungal OTU richness and Shannon diversity are shown for all samples. Mean values for each study population are indicated with larger symbols, and error bars indicate standard deviation. (b) Aitchison distance‐based principal coordinate analysis showing differences in lichen community composition among study groups. (c) Heat map showing significantly differentially abundant lichen groups (represented by their fungal symbiont genera) among study groups and subpopulations. Dashes (−) indicate genera that were not detected in a study group. Differentially abundant genera were defined as genera with an FDR‐corrected *p*‐value < 0.05 and overall abundance > 0.5% across all samples. The LARS reindeer are not represented in panels (b) and (c) due to the low prevalence and relative abundance of lichens in this study group.

We first considered diet‐based differences in the sequencing data, which we interrogated via the lichen fungal and plant ITS amplicons as well as eukaryotic (18S) amplicons assigned to algae. On average, 11.4% ± 16.9% of fungal reads in each sample were assigned to lichen fungal symbionts (Figure S5), while the rest were distributed among lichen parasites, coprophilic fungi, and other environmental fungi (discussed later). Lichen fungal OTU richness and diversity varied significantly among caribou groups: deep‐snow mountain caribou harboured two‐ to three‐fold fewer lichen fungal OTUs than either shallow‐snow mountain caribou or Revelstoke Pen caribou (ANOVA *F*_3_ = 6.41, *p* = 0.001; Figure 2a and Table S2). Only 12 lichen fungal OTUs were detected in the pellet‐fed reindeer from LARS, all at low relative abundance and prevalence (Figure 2a and Table S3), so this population was excluded from subsequent beta‐diversity and differential abundance analysis. The remaining three caribou groups demonstrated clear and significant separation in beta‐diversity analyses (PERMANOVA *F*_2_ = 21.4, *R* ^2^ = 0.46, *p* < 0.001; Figure 2b, Figure S6, and Table S4) and could be discriminated with 97% accuracy in a random forest model trained on their lichen fungal sequencing reads (Table S5).

With respect to taxonomic composition, the arboreal hair lichens *Bryoria* and *Nodobryoria* comprised 62% of all lichen fungal reads in the deep‐snow mountain caribou compared to only 8% in Revelstoke Pen caribou and 0% in shallow‐snow mountain caribou (Figure 2c). The hair lichen genus *Alectoria* was detected only at low relative abundances (0.49%–1.05%) in a subset of deep‐snow subpopulations. In contrast, over 95% of lichen fungal symbiont reads from shallow‐snow mountain caribou were assigned to the terrestrial lichens *Cladonia* (81.5% ± 13.6%) and *Stereocaulon* (14.3% ± 12.8%). These five differentially abundant genera were also implicated in random forest analyses for their ability to differentiate caribou by their study group (Figure 2c and Table S6). Lichen fungal diversity and composition also varied among the deep‐snow caribou subpopulations though to a lesser extent than among the four study groups (Figure S7); this variation was driven primarily by the Central Selkirk subpopulation, which grouped separately from other deep‐snow mountain caribou in beta diversity analyses (Figure 2b, Figure S7, and Table S4) and had fewer than 6% of lichen reads assigned to *Bryoria* and *Nodobryoria* (Figure 2c and Table S6).

Compared to free‐ranging deep‐snow caribou, caribou in the Revelstoke Pen had a significantly higher proportion of reads assigned to *Alectoria* (6.6% ± 10.4%) and three‐fold more reads assigned to the foliose lichens *Platismatia*, *Hypogymnia*, and *Parmelia*, which were otherwise detected at moderate to low relative abundances in only a subset of deep‐snow mountain caribou subpopulations (Figure 2c and Table S6). The hair lichen *Ramalina* was found only in the Revelstoke Pen caribou, and a sequence similarity analysis using the blastn algorithm suggested that this *Ramalina* OTU was *R. thrausta* . *Cladonia* and *Bryoria* accounted for another 9.9% and 7.7%, respectively, of lichen fungal reads from Revelstoke Pen caribou.

#### 3.1.2 Algae

**FIGURE 3:**
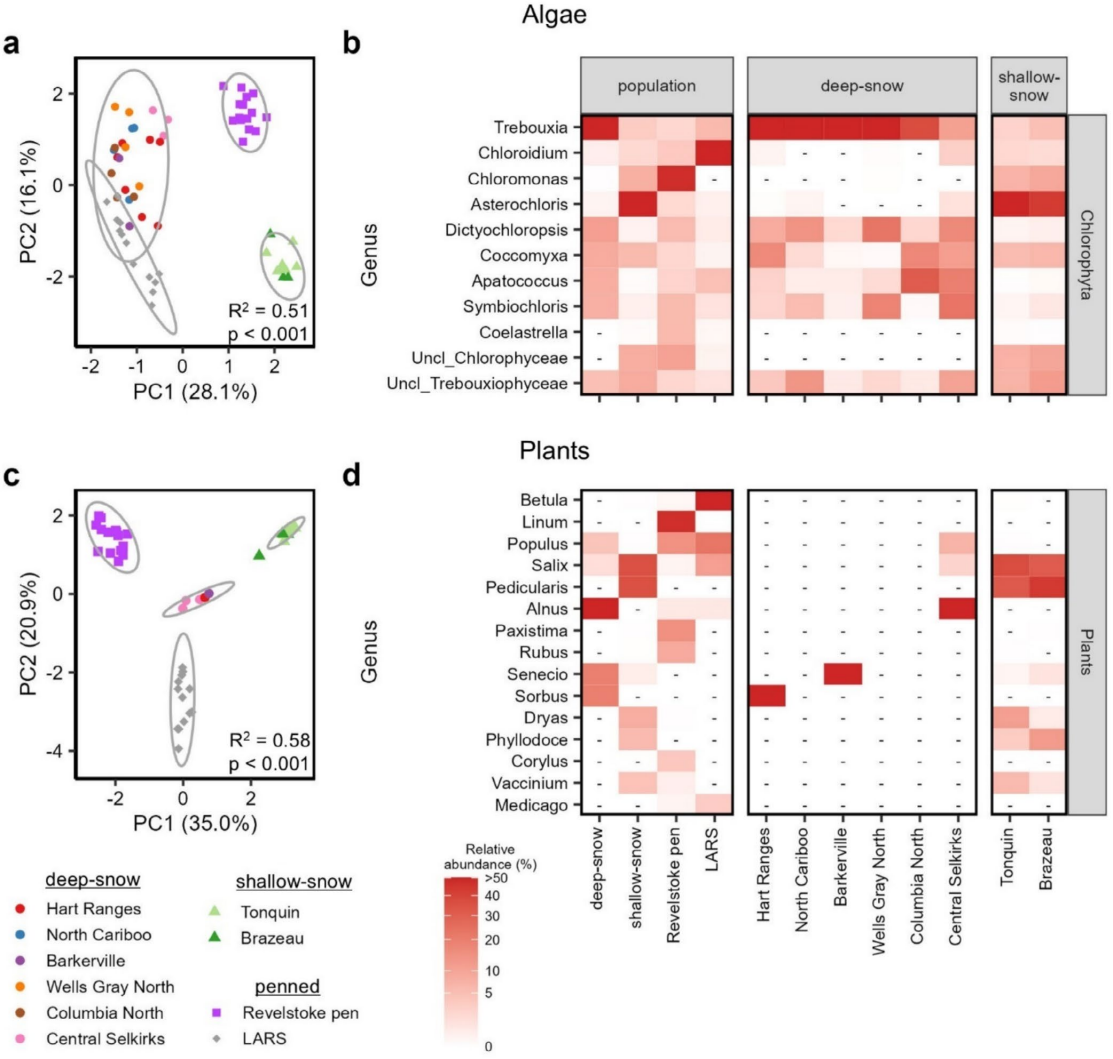
Differences in algae and plant diversity and composition among study groups. (a) Aitchison distance‐based principal coordinate analysis showing differences in algae community composition among study groups. (b) Heat map showing significantly differentially abundant algal genera among study groups and subpopulations. (c) Aitchison distance‐based principal coordinate analysis showing differences in plant community composition among study groups. (d) Heat map showing significantly differentially abundant plant genera among study groups and subpopulations. In (b) and (d), dashes (−) indicate genera that were not detected in a study group. Differentially abundant genera were those with an FDR‐corrected *p*‐value < 0.05 and overall abundance > 0.5% across all samples. Note that sample sizes for the Hart Ranges, Barkerville, and Central Selkirk subpopulations in (d) are 1, 1, and 3, respectively.

The strong differences in lichen fungal composition among study groups were also reflected in the algal reads, which comprised 16.7% of the 18S amplicon reads (Figure S5). Overall, the diversity and composition of algal reads varied significantly among study groups (Figure 3a and Figures S8 and S9; ANOVA *F*_3_ = 80.6, *p* < 0.001; PERMANOVA *F*_3_ = 20.6, *R* ^2^ = 0.507, *p* = 0.001) but not among deep‐snow caribou subpopulations (Tables S2 and S4). *Trebouxia* algae, which are the photosynthetic partner in *Bryoria* lichens (Lindgren et al. 2014), accounted for 56.0% of algal reads from deep‐snow caribou compared to less than 6% of algal reads in the other three groups (Figure 3b and Table S7). Conversely, 54.5% of algal reads for shallow‐snow mountain caribou were assigned to *Asterochloris*, a common photobiont of *Cladonia* and *Stereocaulon* (Skaloud and Peksa 2010) that was detected at less than 2% abundance in other caribou groups (Figure 3b and Table S7). The Chlorophyceae genus *Chloromonas* was not detected in deep‐snow caribou samples but was present at 10.4% abundance among shallow‐snow caribou and 47.7% abundance in Revelstoke Pen caribou. *Chloroidium* was the dominant algal genus detected in the LARS population, comprising 81.4% of all algal reads compared to fewer than 4% in other populations (Figure 3b); this species likely occurs naturally in the LARS enclosure.

#### 3.1.3 Plants

Only five deep‐snow mountain caribou samples produced any plant ITS2 reads. The few plant reads detected in the deep‐snow mountain population were assigned to the genera *Alnus*, *Senecio*, *Populus*, *Salix*, and *Acer* (Figure 3d). Among the remaining three populations, plant OTU richness but not diversity was slightly higher in the Revelstoke pen caribou (Figure S10 and Table S2), and community composition differed significantly (Figure 3c, Figure S10, and Table S4). Most plant reads in the shallow‐snow caribou were assigned to the genera *Pedicularis* (36.1% ± 24.6%), *Salix* (35.8% ± 21.9%), and *Dryas* (8.2% ± 20.3%) (Figure 3d and Table S8), which are typical of alpine ridges in the Alberta Rocky Mountains (Willoughby and Gould 2021). Most plant reads in the Revelstoke Pen caribou were assigned to the genera *Linum* (47.0% ± 25.5%), *Paxistima* (15.0% ± 20.8%), and *Populus* (14.7% ± 21.8%). The captive caribou at LARS consumed primarily *Betula* (56.0% ± 23.6%), *Populus* (22.9% ± 24.7%), and *Salix* (11.4% ± 22.0%) genera (Figure 3d).

### 3.2 Evidence of Unique Microbiome Composition

#### 3.2.1 Bacteria

**FIGURE 4:**
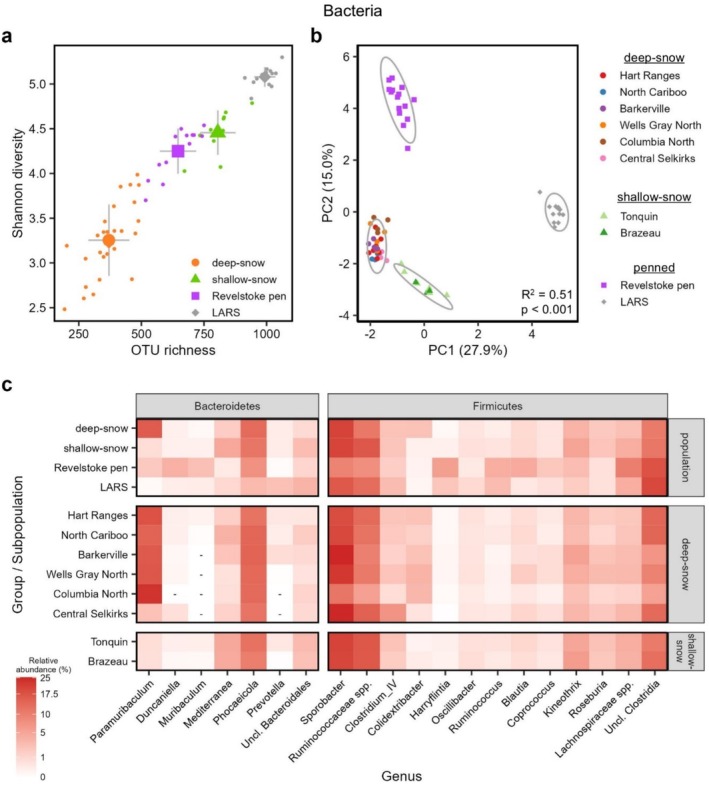
Differences in bacterial microbiome composition among study groups. (a) Bacterial OTU richness and Shannon diversity are shown for all samples. Mean values for each study group are indicated with larger symbols, and error bars indicate standard deviation. (b) Aitchison distance‐based principal coordinate analysis showing differences in bacterial community composition among study groups. (c) Heat map showing significantly differentially abundant bacterial genera among study groups and subpopulations. Dashes (−) indicate genera that were not detected in a study group. Differentially abundant genera were defined as genera with an FDR‐corrected *p*‐value < 0.05 and overall abundance > 0.5% across all samples.

We evaluated how diet‐based differences among caribou groups correlated with gut bacterial microbiome composition as revealed through the 16S amplicon data. Overall, the gut microbiome across all samples was dominated by Firmicutes and Bacteroidetes (> 97% of all reads), with most reads assigned to the families Ruminococcaceae, Lachnospiraceae, and Bacteroidaceae (Figure S11). We also detected a small population of methanogenic archaea (Figure S11). With respect to our study objectives, we found that the gut microbiome of deep‐snow caribou was significantly less species‐rich (ANOVA *F*_3_ = 250.7, *p* < 0.001) and diverse (ANOVA *F*_3_ = 118.0, *p* < 0.001) than that of any other caribou population we sampled, harbouring 51% and 43% fewer bacterial OTUs than the shallow‐snow and Revelstoke Pen caribou, respectively (Figure 4a and Table S2). The LARS population had the most species‐rich microbiome (Figure 4a). As with diet, gut microbiome composition differed significantly among all four study populations (Figure 4b and Figure S12; PERMANOVA *F*_3_ = 21.2, *R* ^2^ = 0.506, *p* = 0.001) and perfectly discriminated among study populations in random forest models (Table S5). Bacterial alpha and beta diversity also varied significantly among deep‐snow mountain caribou subpopulations, though to a lesser extent than among the four study groups (Table S4 and Figure S13).

Twenty‐four bacterial genera were significantly differentially abundant among study groups. Most strikingly, *Paramuribaculum* was 9‐ to 24‐fold more relatively abundant in deep‐snow caribou than in the shallow‐snow or Revelstoke Pen caribou and nearly absent from the LARS population (Figure 4c and Table S9). The genera *Phocaeicola* and *Sporobacter* were two‐fold more relatively abundant in the free‐ranging relative to the penned and semi‐domesticated populations (Figure 4c and Table S9); *Ruminococcus* and other unclassified Ruminococcaceae were 1.3‐fold more relatively abundant in LARS caribou, and *Blautia* was five‐fold more abundant in Revelstoke Pen caribou. Other genera implicated in the random forest analyses included *Subdoligranulum*, which was present in all Revelstoke Pen caribou but undetected in all other groups, and several genera that were only present in the LARS reindeer (Table S9). Few bacterial genera were differentially abundant among deep‐snow mountain subpopulations, with the notable exception of *Paramuribaculum* being less relatively abundant in the Central Selkirk subpopulation (Figure 4c).

#### 3.2.2 Protists

Across all samples, the protist community in the gut microbiome was predominantly composed of *Entamoeba* (38.1% ± 27.5%), which did not vary in relative abundance among our study populations and various Apicomplexan groups (34.6% ± 33.8%) (Figure S14). Although Apicomplexans were three‐fold more relatively abundant in deep‐snow mountain caribou than in other populations, two‐thirds of the sequencing reads assigned to Apicomplexa could not be identified below the class level (Table S10), limiting our inferential power about the potential role of the protist microbiota in our study populations. Most notably, putative Conoidasida comprised 43.0% of reads from deep‐snow caribou compared to fewer than 5% of reads from other study groups (Figure S14 and Table S10). We otherwise observed that the protist microbiota of the two free‐ranging caribou groups remained distinct from the Revelstoke Pen caribou and LARS reindeer in terms of both alpha‐ and beta‐diversity (Figure S14). Specifically, protist alpha diversity was two‐fold higher in the two captive groups (Figure S14 and Table S2), which clustered separately from free‐ranging caribou and from each other in beta‐diversity analysis (Figure S14 and Table S4). More protist OTUs were detected in shallow‐snow than deep‐snow caribou, but these two groups did not differ significantly in community composition (Figure S14 and Table S2).

### 3.3 Other Amplicon Signatures

In addition to diet‐ and microbiome‐based signatures, we detected several non‐lichen fungal genera that represented over 70% of the fungal ITS2 sequencing reads. These taxa presumably represent a mixture of environmental fungi, foraging bycatch, and members of the gut mycobiome. For example, the coprophilic genus *Thelebolus* was abundant in most samples and accounted for 27% of all fungal ITS reads (Figure S15 and Table S11). The fungus *Lichenoconium*, known to parasitize *Nodobryoria* (Cole and Hawksworth 2004), was significantly more relatively abundant in deep‐snow mountain caribou than in other study groups (22.0% vs. < 5%) (Figure S15 and Table S11). Other relatively abundant fungi in the sample included the family Myxotrichaceae, which was detected at moderate relative abundances in deep‐snow and penned caribou, and the saprotrophic genus *Troposporella*, which was most relatively abundant in shallow‐snow caribou (Figure S15 and Table S11).

### 3.4 Diet as a Predictor of Microbiome Composition

**FIGURE 5:**
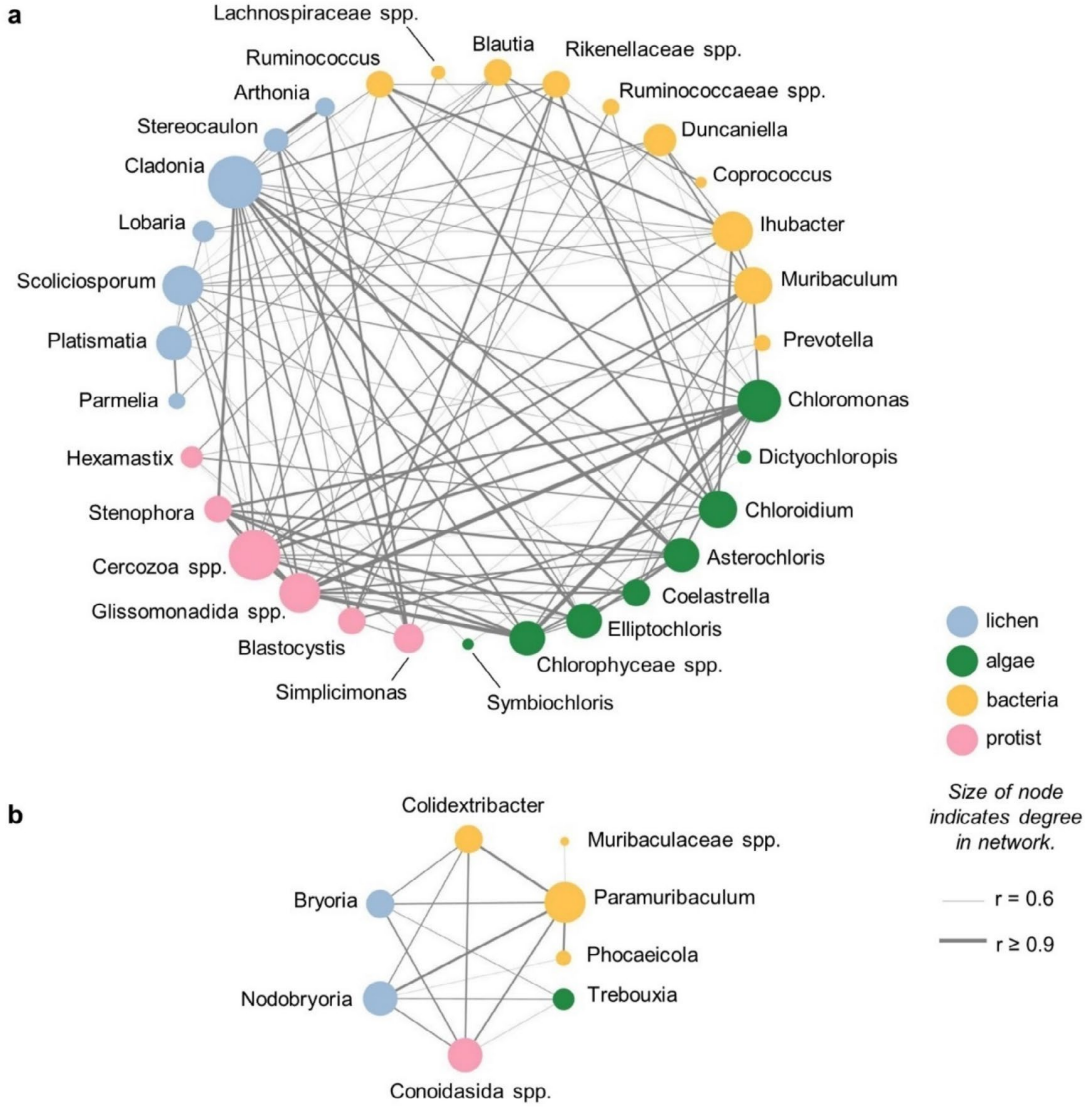
All‐amplicon co‐occurrence network. We generated an amplicon co‐occurrence network for all lichen, algal, bacterial, and protist genera present in > 4 samples with a mean relative abundance > 0.5%. Significant co‐occurrences were defined based on Spearman's correlations between amplicon relative abundances. Only connections with Spearman's *r* > 0.6 and FDR‐adjusted *p*‐value < 0.01 are shown. This co‐occurrence analysis resulted in two separate networks: The first, (a) contains indicator species for shallow‐snow and penned caribou as well as the semi‐domesticated reindeer from LARS; the second, (b) largely represents deep‐snow caribou.

Consistent with the strong separation among study populations for all amplicons we tested, our RDA analyses showed that gut microbiome composition (bacteria or protists) across our four study populations was well explained by all three dietary components (lichens, algae, or plants) (*p* < 0.05 for all comparisons; Table S12). Moreover, our co‐occurrence analysis among bacterial/archaeal, protist, algal, and lichen OTUs identified two disconnected communities of OTUs representing different diets and microbiome composition: a smaller community that was characteristic of deep‐snow mountain caribou and a second, larger community that was characteristic of shallow‐snow mountain caribou (Figure 5, Tables S13 and S14). In the first community, the relative abundance of *Bryoria* and *Nodobryoria* among lichen reads was significantly intercorrelated with the relative abundances of *Trebouxia* algae, the bacterial genera *Paramuribaculum*, *Colidextribacter*, and *Phocaeicola*, and the unclassified Conoidasida. In the second community, *Cladonia* and *Platismatia* were the main lichen hubs and *Chloromonas* and Chlorophyceae spp. were the main algal hubs; *Ihubacter*, *Muribaculum*, and Cercozoa spp. were the most intercorrelated genera from the microbiome.

## 4 Discussion

When organisms have restricted diets, whether this occurs within a species or, as for caribou, among populations or ecotypes, the chances that habitat loss will negatively impact populations are increased (Slatyer et al. 2013). The continued decline of Canada's endangered deep‐snow and shallow‐snow mountain caribou populations calls for the use of a breadth of information and tools to support ongoing efforts for population recovery. Experience has shown the importance of animal health in successful endangered species recovery programs (Beckmann et al. 2022), and recent evidence has suggested that preserving natural diets and maintaining a healthy gut microbiome may enhance conservation efforts (Trevelline et al. 2019; West et al. 2019). These considerations may be especially important for caribou, where specialised diet and habitat requirements that vary among populations make each ecotype especially vulnerable to environmental change (Clavel et al. 2011). Here, we used DNA metabarcoding of faecal pellets to explore variation in caribou winter diets and microbiomes, and our results support three main conclusions about deep‐snow caribou ecology with implications for captive or *ex‐situ* rearing programs: (a) we confirmed the historical characterisations of a highly distinct, arboreal lichen‐based winter diet for deep‐snow caribou, which (b) corresponds to a unique gut microbiome with additional variation among subpopulations of deep‐snow caribou, that (c) appears to have been altered in the maternity penned caribou sampled for this study, despite having been in the pen for only a short time.

### 4.1 Winter Diet and Gut Microbiome of Free‐Ranging Caribou

Overall, our results from diet‐focused amplicons are consistent with our prediction that the winter diet and gut microbiome of deep‐snow mountain caribou are distinct and less diverse from that of shallow‐snow mountain caribou. As shown in previous studies of both free‐ranging and tame subpopulations (Cichowski 1993; Denryter et al. 2017), shallow‐snow mountain caribou consumed terrestrial lichens along with some willows, ericoid shrubs, and mountain‐avens; for lichens, these caribou appeared to overwhelmingly prefer *Cladonia* spp. and, to a lesser extent, *Stereocaulon*, with more limited consumption of *Cetraria* (Figure 2). Conversely, deep‐snow mountain caribou specialised almost exclusively on arboreal hair lichens; specifically, fungal data showed a strong preference for the darkly pigmented, melanin‐rich *Bryoria* and *Nodobryoria* lichens over usnic acid‐rich *Alectoria* lichens. *Bryoria* is more abundant than *Alectoria* in the treeline habitats that deep‐snow caribou occupy from January to April (Apps et al. 2001; Edwards et al. 1960), but we suspect that the sequencing read ratios reflect feeding selectivity rather than forage availability because both observational and cafeteria‐style trials have demonstrated that caribou prefer *Bryoria* and *Nodobryoria* over *Alectoria* by a wide margin (Rominger and Robbins 1996; Rominger et al. 1996). This preference may stem from an avoidance of usnic acid, a visibly pigmented metabolite that has been reported to be toxic for elk ( *Cervus canadensis* ) (Cook et al. 2007; Roach et al. 2006) and sheep ( *vis aries* ) (Dailey et al. 2008) and is absent in *Bryoria* and *Nodobryoria*. Another hypothesis is that the low protein content of *Alectoria* compared to *Bryoria* causes avoidance by caribou (Rominger et al. 1996) and mule deer (Robbins 1987). Lack of awareness of caribou forage preference might be the reason that *Alectoria* was accepted as a volunteer‐collected feed for maternity‐penned caribou (Legebokow 2016), a practice confirmed by our finding that the Revelstoke Pen was the only sample group to contain a significant amount of *Alectoria* reads.

While our data confirm avoidance of *Alectoria* lichens, we also found that deep‐snow caribou do consume other arboreal lichens that lack usnic acid but have received less attention in past studies. These other dietary components include representatives of two different lichen morphological groups: foliose lichens and crustose lichens, both of which were detected at moderate relative abundances. The most prominent leaf lichen genera in the deep‐snow caribou data were *Hypogymnia, Parmelia*, *Platismatia*, and *Lobaria*. All four genera are common and produce substantial biomass in forests used by deep‐snow caribou; in addition, *Platismatia* has previously been detected in rumen samples (Antifeau 1987), and studies have observed *Lobaria* being consumed at feeding sites (Edwards and Ritcey 1960). The widespread occurrence of the crustose genus *Lecanora* among deep‐snow caribou suggests that foraging caribou strip the bark off twigs, where lichens such as *Lecanora circumborealis* commonly co‐occur with *Bryoria* (an interpretation consistent with the occurrence of woody plant parts in rumen contents; Antifeau 1987). As expected, few sequencing reads from deep‐snow mountain caribou were assigned to plant forage and terrestrial lichens because these forage types are largely unavailable and/or do not grow underneath the deep snowpack in their winter range.

The composition of the caribou gut microbiome closely followed diet signatures: diet signatures explained microbiome composition in RDA analysis, and deep‐snow mountain caribou were distinct enough in diet and microbiome composition that they formed a separate network in our co‐occurrence network analysis (Figure 5). Overall, we expected high abundances of fermentative taxa essential for the digestion of plant‐ and lichen‐based diets, which was supported by the general dominance of Ruminococcaceae species in the gut microbiome. Our findings align with previous reports from Norwegian reindeer (*R. t. tarandus*) (Sundset et al. 2009), Svalbard reindeer (*R. t. platyrhynchus*) (Zielińska et al. 2016), and several other ruminants (de Jonge et al. 2022; Henderson et al. 2015). Deep‐snow mountain caribou were uniquely distinguished by a high relative abundance of the bacteria *Paramuribaculum*, and, to a lesser extent, by higher abundances of *Colidextribacter* and *Phocaeicola*. *Paramuribaculum* is a recently described genus of Muribaculaceae that is characterised by its expression of genes related to the degradation of complex carbohydrates such as chitin or cellulose (Lagkouvardos et al. 2019); *Colidextribacter* and *Phocaeicola* have similarly been associated with carbohydrate fermentation and short‐chain fatty acid production (Liu et al. 2022; Lück and Deppenmeier 2022). The latter is more abundant in free‐grazing livestock with high‐fibre diets (Wang et al. 2020, 2019), indicating their potential importance for the degradation of complex lichen polysaccharides.

We suspect that these unique microbiomes may arise in part because of the distinct polysaccharide composition of arboreal and terrestrial lichens, though we are unable to directly evaluate the functional potential of the microbiome. As we expected, the winter diet of deep‐snow caribou is dominated by lichens with mostly β‐glucans in their cell walls (e.g., *Bryoria*), while shallow‐snow caribou prefer lichens with α‐glucans (e.g., *Cladonia* and *Stereocaulon*), albeit with some overlap between these caribou and glucan types (e.g., *Cetraria* lichens consumed by shallow‐snow caribou contain some β‐glucans) (Olafsdottir and Ingólfsdottir 2001). Although we found strong correlations between the dietary abundance of these lichens and particular microbiome components (Figure 5), our data do not allow us to confirm that the unique microbiome of deep‐snow caribou is driven by specialisation of enzymatic machinery to digest β‐glucans. This is because amplicon data do not provide the whole‐genome information required to infer whether genera such as *Paramuribaculum* code for the enzymes required for the degradation of specific polysaccharide linkages (Djemiel et al. 2022). The one published genome available for *Paramuribaculum* (Low et al. 2023) contains little evidence of β‐glucan specialisation (see Figure S16); nevertheless, the enzymatic potential of this family of bacteria is poorly characterised and may vary among species or strains (Lagkouvardos et al. 2019). The uniquely high relative abundance of *Paramuribaculum* in deep‐snow caribou microbiomes suggests that this bacterium could be an important target for future research and monitoring efforts.

### 4.2 Commercial Pelleted Feed and Maternity Penning

As predicted, the deep‐snow caribou in the Revelstoke maternity pen exhibited dietary signatures and gut microbiome composition that were sharply different from those of their free‐ranging counterparts. Although the penned caribou still consumed *Bryoria*, a much greater proportion of lichen fungal symbiont fungal reads in the penned caribou were assigned to foliose lichens and to *Alectoria* (Figure 2). This dietary shift could be due to forage availability in the pen, lichen feed provided by volunteers, or unknown factors associated with penning and commercial feed that may have altered feeding preferences. Notably, penned caribou also appeared to have consumed *Ramalina* lichens, which were undetected in other study groups and contain usnic acid (Dailey et al. 2008; Roach et al. 2006). This result is striking as *Ramalina* lichens are rare to absent in the forests where the pen was located (T. Spribille, personal observation). One possible explanation for the occurrence of *Ramalina* DNA in Revelstoke Pen faecal pellets is that *Ramalina thrausta* could have been mistakenly gathered by volunteers intending to collect *Alectoria sarmentosa* as caribou feed, as it can be easily confused with *Alectoria* and is common in nearby lowlands.

Penned deep‐snow caribou also appeared to consume more plants than free‐ranging deep‐snow caribou, as only five samples from free‐ranging individuals produced detectable plant sequences (Figure 3). Interestingly, almost half of the plant sequences in the Revelstoke pen population were assigned to flax (*Linum* spp.), which does not grow in the pen area. The presence of flax is almost certainly a signature of the commercial feed provided to the caribou, which is 3.5% flax by weight (Wetaskiwin Coop Assoc. Ltd.). Other abundant plant sequences in the Revelstoke pen were assigned to *Paxistima* and *Populus*, which are common forages in that pen area. Signatures of the commercial feed provided at LARS (which contains barley, alfalfa, and corn) could similarly be detected in the LARS reindeer though these sequences were less relatively abundant than those of native plants in the LARS pen area. Although the abundance of plant sequences in the Revelstoke pen caribou may be due to a lack of available lichen forage, it may also be because faecal samples from these caribou were collected during spring green‐up, whereas the samples from free‐ranging caribou were collected in late winter. This difference in collection schedules was necessary to collect free‐ranging samples during the GPS‐collaring season and to accommodate the timing of the transition to a pellet diet for the penned animals. However, this timing makes it possible that penned caribou consumed more plants because more plants with greater quantities of protein were available as natural forage in pens at the time of sample collection.

The gut microbiome of penned deep‐snow caribou was sharply different from that of free‐ranging deep‐snow caribou, though still more similar to free‐ranging conspecifics than to the semi‐domesticated LARS reindeer in distance‐based beta diversity analysis (Figure 4). Most notably, penned caribou harboured significantly fewer *Paramuribaculum* but significantly more *Muribaculum* and *Duncaniella*. These two genera are closely related to *Paramuribaculum* and similarly known for degrading complex polysaccharides (Lagkouvardos et al. 2019), making it difficult to comment on whether or how these taxonomic differences in microbiome composition could be reflected in microbiome function or enzymatic machinery. Indeed, publicly available genomes from representative species of these three genera (Beresford‐Jones et al. 2022; Miyake et al. 2019; Miyake et al. 2020a, 2020b) show little evidence of metabolic specialisation among their associated families of carbohydrate‐degrading enzymes [i.e., CAZymes (Cantarel et al. 2009)] (see Figure S16). However, those available genomes do not come from species found in the caribou gut, and microbial functional potential varies widely even among species or strains from the same genus (Van Rossum et al. 2020). Determining whether the altered microbiome of penned caribou represents some loss or gain of metabolic function relevant to digestion or animal health will therefore require more detailed functional or metagenomic analyses.

### 4.3 Limitations

Although our results were strikingly consistent with our predictions that (a) deep snow caribou would exhibit narrower and more arboreal lichen‐based winter diets than shallow snow caribou, (b) the microbiome would reflect those dietary differences, and (c) microbiomes would be substantially disrupted by pelleted feed, our sampling design imposes at least four limits on the generality of these findings. First, we focused on winter diets because of the known differences in winter foraging habits among caribou subpopulations, but diet and the associated microbiome can vary seasonally. For this reason, faecal samples collected in winter for free‐ranging caribou may not be comparable to faeces collected in spring for penned caribou; in addition, deep‐ and shallow‐snow caribou have more similar foraging habits in their summer ranges (Webber et al. 2022). Seasonal variation in microbiome composition has been documented in Russian reindeer, and though this variation is relatively minor (Ilina et al. 2021; Yildirim et al. 2021), future work should collect samples from throughout the year and compare them within and among seasons. Second, our study design did not allow us to determine whether caribou regain their wild‐type microbiome as they are transitioned back to natural forage prior to release; more extensive sampling before, during, and after the capture period would answer this important question. Third, while our sample sizes were appropriate for assessing variation among our four study groups, we were unable to assess the potential role of other known influences on diet and/or microbiome composition [e.g., sex, age, or species identity (Macke et al. 2017)] or to robustly characterise within‐group variation, especially for the anomalous Central Selkirk subpopulation. A fourth limitation is that we had samples from only one maternity pen; future studies that compare multiple samples from multiple pen sites will be better able to determine the effects of pelleted diets on microbiome composition and function as well as the extent to which microbiome composition in captivity is related to animal health or post‐release fitness.

There are also some limitations specific to DNA‐based metabarcoding approaches. For example, the survivability of dietary DNA during digestion may vary among forage types, which would bias our estimates of diet composition. Furthermore, the percentage of fungal reads assigned to lichens varied significantly among individuals and was generally low, whereas coprophilous fungi were present with comparatively high relative abundances despite samples being collected shortly after deposition. In this case, we suspect that fungal DNA counts were skewed towards organisms adapted to survive or even benefit from digestion, and we attempted to mitigate this effect by analysing lichen reads separately from those of other fungi. Finally, deep‐snow mountain caribou produced fewer reads for most amplicons, which may limit our inferential power. We attempted to compensate for this by extrapolating richness and diversity estimates from rarefaction curves, following best practices for the analysis of compositional data (Gloor et al. 2017), and comparing our results to rarefied data to confirm that our conclusions are robust to different analysis methods.

## 5 Stewardship Implications

Despite the limitations described above, our results demonstrate the potential role of molecular evidence in conservation planning for mountain caribou with principles that generalise to other species. Our study is the first to demonstrate that, at least in late winter, deep‐snow mountain caribou possess a distinct diet and gut microbiome that overlaps little with that of their nearest neighbours, the shallow‐snow mountain caribou. We additionally demonstrated that this specialised gut microbiome can be altered when caribou are held in maternity pens and provided with commercial feed. Our results raise the important question of whether a transition off natural diets has consequences for caribou health, body condition, or fitness, which will require further research. Nevertheless, the need to recognise the potential vulnerability of dietary specialists, and their corresponding sensitivity to management efforts, underscores three precautionary implications that our results carry for future conservation efforts.

First, if maternal penning or other *ex situ* programs (e.g., captive breeding) are considered for caribou recovery, they could benefit from careful consideration of population‐specific natural diets. For example, the collection of lichens that are preferred by target populations (e.g., *Bryoria* over *Alectoria*) will require collectors to be adequately trained in lichen identification and habitat associations. If diets are supplemented with commercial feed, the transition to pellet feed could be made easier with a pellet composition that mimics natural dietary components as closely as possible; for example, feed pellets could be formulated to contain the same type of polysaccharides as natural forage. These same considerations could also inform in situ supplemental feeding experiments; for example, the Kennedy Siding subpopulation of shallow‐snow mountain caribou is supplemented with commercial pelleted feed in the wild for 2 months in the late fall, an intervention that has had positive effects on body condition, survival, and abundance (Heard and Zimmerman 2021; Lamb et al. 2024) but unknown effects on gut microbiome composition.

Given the strong differences we observed between deep‐snow and maternity‐penned caribou, a second implication of our results is the value of consistent and longer‐term monitoring of the caribou microbiome, especially before, during, and after periods of captivity, as an additional metric that could guide stewardship decisions. Faecal samples can be collected regularly and non‐invasively, especially in captive populations during and after dietary transitions, and several commercial laboratories specialise in processing microbiome samples and data at accessible costs. In combination with other metrics of body condition, regular information about microbiome composition could support dietary adjustments to maintain the microbiome of temporarily captive animals (Dallas and Warne 2023). Longer‐term monitoring studies could also identify the optimal rate for transitioning diets to or from pelleted feed or the minimum necessary proportion of natural lichens in a pelleted diet as well as the extent to which gut microbiome composition is related to health or post‐release fitness. Indeed, pre‐ and post‐release microbiome surveillance has successfully been employed as part of *ex situ* interventions for other endangered species, including pre‐release diet training to promote a wild‐type microbiome (Blyton et al. 2023; Chong et al. 2019; Yang et al. 2020).

Finally, the narrow range of lichens preferred by deep‐snow caribou reinforces the broader message that maintaining free‐ranging in situ caribou populations will require greater protection of the old‐growth forest habitat that supports high quantities of the specific lichen species that these endangered animals consume (Holt 1977; Serrouya, Dickie, et al. 2021). For example, maintaining healthy, high‐biomass *Bryoria* loadings requires forest stands that are well‐ventilated (Gauslaa 2023; Goward 1998) a feature that is related to attributes of stand age, structure, and slope position (Goward 1998; Goward et al. 2022). The current protections for these old‐growth forests have not been sufficient to prevent caribou decline (Environment Canada 2014). We suggest that molecular methods could be combined with models of forage quality (Bishop et al. 2009) to estimate the amount of specific habitat types needed to support self‐sustaining populations of deep‐snow caribou and guide evidence‐based restrictions on human activity in these areas.

Each of the three implications we outline above for caribou conservation—careful matching of the wild diet in the feed of captive animals, monitoring the effect of diet transitions via the microbiome, and ensuring adequate habitat protection or restoration for reintroduced animals—applies to the growing number of threatened and endangered species for which unabated habitat loss and degradation have created a need for *ex situ* conservation (Myers et al. 2000; Slatyer et al. 2013). As a precautionary principle, we therefore suggest that information about population‐ or ecotype‐specific diet and microbiome composition should be considered when designing or implementing programmes that require providing supplementary feed to endangered animals. Seeking such actionable, multidisciplinary information is a necessary component of adaptive management to achieve conservation goals in forested landscapes (Lindenmayer et al. 2000).

## Author Contributions

S.S., C.C.S.C., L.S., and T.S. designed the study; C.C.S.C., L.S., and T.S. acquired funding, and R.S., L.N., and H.S. collected and/or provided caribou faecal samples and valuable perspectives on caribou ecology and management. S.S. performed laboratory work, analysed the data with support from T.S., and led the writing of the manuscript. All authors contributed critical feedback on the analysis and manuscript text, and all authors have read and agreed to the final version of the manuscript.

## Disclosure

A research collaboration was developed with scientists from the various agencies that contributed samples for this study, and all collaborators are included as co‐authors. Our data and results are available on public databases, as described above, and our research supports the conservation of a threatened Canadian species.

## Conflicts of Interest

The authors declare no conflicts of interest.

## Supporting information


Data S1.

Table S1.

## Data Availability

All sequencing data produced for this manuscript have been deposited in the NCBI Short Read Archive (SRA) under BioProject accession number PRJNA1067083. Sample metadata and the R codes required to reproduce all analyses and figures are available at https://doi.org/10.5281/zenodo.14862504.
