# Supplementary material for: Endangered Deep‐Snow Mountain Caribou Have a Distinct Winter Diet and Gut Microbiome That May Be Altered by Maternal Penning
Source: Mol Ecol. 2025 May 7;34(11):e17783. doi: 10.1111/mec.17783 (PMC12100581; doi:10.1111/mec.17783)
Supplement: Supplementary file 1 — Data S1. [file MEC-34-e17783-s001.pdf]

MOLECULAR ECOLOGY

Supplemental Information for:

Endangered deep-snow mountain caribou have a distinct winter diet and gut microbiome that may be altered by maternal penning

Scott Sugden, Robert Serrouya, Lalenia Neufeld, Helen Schwantje, Colleen Cassady St. Clair, Lisa Stein, Toby Spribille

Supplementary methods **2**

References **8**

Supplementary tables **10**

Sequencing overview **13**

Evidence of unique diets **18**

Evidence of unique microbiome composition **23**

Other amplicon signatures **27**

Muribaculaceae genome analysis **28**

SUPPLEMENTARY METHODS

OTU generation. After we removed primers from sequencing reads using cutadapt [1], all downstream 16S rRNA gene sequence analysis was performed in the R package *dada2* [2]. For the 16S rRNA, 18S rRNA, and fungal ITS2 amplicons, we truncated forward and reverse reads at 220 bp and 180 bp, respectively; because the range of expected lengths for the plant ITS2 amplicons is slightly longer (**Table S1**), we truncated forward and reverse plant ITS2 reads at 260 bp and 220 bp, respectively. Low-quality reads were then removed from the data using the *dada2* default filtering parameters. We merged paired-end reads and determined exact sequence variants using the pooled inference procedure in *dada2*. Amplicon sequences were then clustered into operational taxonomic units (OTUs) at 97% sequence identity using the --cluster-size command in *vsearch* [3]. We used an OTU-based clustering approach, rather than retaining exact sequence variants, to reduce the risk of either overrepresenting the number of taxa detected in each sample or producing other artifacts related to within-species variation in 16S rRNA gene sequences [4, 5]. Because the amplicon produced by the 16S rRNA gene primers has a tight length distribution around 372 bp, we only retained bacterial OTUs with a length of 369-375 bp. This exclusion criteria removed 4.65% of the sequencing reads; of these, 51.7% were assigned to methanogens and another 29.2% were assigned to unclassified bacteria or unclassified Firmicutes. No size trimming was performed for the 18S, fungal ITS2, or plant ITS2 amplicons.

Taxonomic annotation. To assign taxonomy to the 16S, 18S, and fungal ITS2 reads, OTUs were aligned against taxa in the RDP v18 [6], SILVA v138 [7], and UNITE v9.0 [8] databases, respectively, using the naïve Bayesian classifier method implemented in *dada2* [9]. For the 16S reads, we removed OTUs that were identified as chloroplasts (n=5, 0.012% of reads), plants (n=1), and archaea (n=10, 2.4% of reads). The ten archaeal OTUs came from two methanogenic genera and were considered separately for our analysis (see **Fig. S11**). We also performed a BLAST search of bacterial OTUs that did not have a phylum-level annotation (n=24, 0.063% of reads), but because the closest hits for these OTUs were unidentified, uncultured bacteria, all 24 OTUs were removed from analysis.

Because the publicly available SILVA 18S database [7] contains several errors in its higher-level taxonomic classifications (e.g., cases where genus names are listed as species or family names), we used a curated version provided by Patrick Gagné (Natural Resources

Canada). We made further corrections to this database to ensure that higher-level taxonomic ranks were consistent throughout the 18S data; the R code outlining these changes is provided in the GitHub repository associated with this manuscript (see **data availability**). As with the 16S OTUs, we performed a BLAST search of the 173 18S OTUs (5.06% of reads) that did not have a phylum-level annotation. We discarded 8 OTUs with no significant matches and then used the BLAST results to provide more complete annotations for the remaining 165 OTUs. We also discarded OTUs with no further classification beyond "eukaryote" (n=27, 0.14% of reads) and OTUs that were assigned to Mammalia (n=16, 8.15% of reads).

After aligning fungal ITS2 OTUs against the UNITE database, 342 OTUs (representing 4.47% of reads) lacked a phylum-level classification. We used a BLAST search to obtain annotations for these OTUs; one OTU was discarded due to no significant BLAST similarity, and another 196 OTUs (3.53% of reads) were discarded because they were assigned to plants or algae. The remaining 145 OTUs did not have significant (>97% identity) BLAST hits beyond "unclassified fungi" and were therefore retained as "unclassified fungi" for analysis. We additionally reviewed the fungal ITS2 annotations by comparing taxonomic assignments from the UNITE database with the results of BLAST searches for both high-abundance amplicons and lichen fungal symbionts. In select cases, we used the results of these BLAST searches to correct the UNITE-based annotations: specifically, 6 of the 7 OTUs that were annotated as *Dothiorella* by the UNITE database were reclassified as *Lichenoconium*; OTUs that were annotated as *Austrolecia* (n=3) or *Rhizoplaca* (n=6) were reclassified as *Lecanora*; and OTUs that were annotated as *Phylliscum* (n=3) were reclassified as *Rhinocladiella*.

Because there is no publicly available plant ITS2 database, we generated a taxonomy table for all 327 plant ITS2 OTUs using a BLAST search. For each OTU, we retained the BLAST results with the highest bit score. If multiple hits had the same bit score, we classified the OTU to the lowest taxonomic level at which all top-ranking hits agreed. We then removed two OTUs classified as algae (0.0024% of reads).

Sequencing controls. Between our two negative control samples, we detected a total of two 16S OTUs and one 18S OTU (**Fig. S1**). No fungal or plant reads were detected in either negative control sample, and none of these contaminants were abundant or prevalent in our

experimental data (**Fig. S1**). All three OTUs detected in the negative control samples were removed from our experimental samples.

We also sequenced the ZymoBIOMICS Microbial Community DNA Standard alongside our experimental samples. We confirmed the accuracy of our mock community by aligning our mock community OTU sequences with known reference sequences using MAFFT [10] and comparing genus-level relative abundances to expected values (**Fig. S2**). We further determined that the Measurement Integrity Quotient (MIQ) [11] for our mock community was 74%, suggesting reasonable agreement between our results and expected values.

Data filtering. After removing control samples from the analysis, we further subset our data to remove low-abundance or potentially spurious OTUs. We first removed samples with fewer than 2,000 16S reads (n=1) and samples with fewer than 200 plant ITS2 reads (n=5) (**Fig. S1**). Note that samples with low read counts for one amplicon were still retained in the analyses of other amplicons. For each of the four amplicons, we then removed all OTUs with a relative abundance <0.001% across the data set. This led to the removal of 1488, 808, 1231, and 125 OTUs from the 16S, 18S, fungal ITS2, and plant ITS2 data, respectively.

Phylogenetic tree construction. We generated phylogenetic trees for the remaining OTUs following the procedures described by Callahan et al. [12]. For each amplicon, we first performed a multiple-sequence alignment using *DECIPHER* [13]. We then used *phangorn* [14] to generate a generalized, time-reversible phylogenetic tree with Gamma rate variation.

Identification of lichens, algae, and protists. We separated lichen fungal symbionts from the fungal ITS2 amplicons by identifying taxa considered as such by either Lücking et al. [15] or FUNGuild [16]. We then manually reviewed the list of lichen fungal symbionts and removed *Sarea*, which is not considered a lichen-forming fungus by most modern authors [17]. Algal (n=82) and protist (n=213) OTUs were separated from the 18S data based on the taxonomic assignments obtained from the SILVA database; these groups accounted for 16.7% and 50.9% of the 18S data, respectively. The remaining 18S OTUs were assigned to Animalia (n=30, 3.7% of reads; mostly tardigrades and nematodes), Fungi (n=155, 7.7% of reads), and Plantae (n=55, 21.0% of reads) (**Fig. S5**).

Because fungal and plant sequences were targeted more directly with the fungal ITS2 and plant ITS2 primers, which are more sensitive for these groups, we did not use these remaining 18S sequences for any analyses. A cursory comparison of the 18S and ITS2 results supported this approach: in addition to providing more reads for analysis, the ITS2 primer pairs recovered more taxa than the 18S primers (467 fungal genera and 95 plant genera compared to only 72 and 23, respectively). Nevertheless, the 18S primers did capture select groups not detected by the ITS2 primers: most notably, two basidiomycete yeasts (*Meyerozyma* and *Derxomyces*) and the anaerobic fungus *Orpinomyces* together accounted for 37.1% of all 18S reads assigned to fungi, and the moss genus *Parisia* accounted for 11.3% of all 18S reads assigned to plants. Other taxa Given these results, we do not believe the omission of taxa undetected with the ITS2 primers compromises our downstream data analysis.

Downstream analyses. All estimates of alpha diversity (e.g., taxa richness or Shannon diversity) for each amplicon or amplicon subset were calculated on unrarefied count data using *iNext* [18], as described in the main text. All beta-diversity analyses were performed using unrarefied, centered log-ratio (CLR)-transformed feature tables, as this approach best accounts for the compositional nature of sequencing data (i.e., the fact that sequencing data only provides information on relative abundances as the total number of reads retrieved varies among samples) [19]. Ordinations and PERMANOVAs were calculated from CLR-transformed data using the Euclidean distance; this metric (the Euclidean distance applied to CLR-transformed data) is also known as the Aitchison distance. For differential abundance analysis, the *ALDEx2* algorithm [20] similarly accounts for compositionality by implementing a CLR transform prior to identifying significant differences in relative abundances.

To ensure that our biological results were robust to our decision to use unrarefied, CLR-transformed data, we performed all analyses in parallel on rarefied data. For each amplicon or amplicon subset (i.e., lichens, algae, or protists), we averaged OTU abundances across 1,000 rarefactions to the minimum library size in each data set. Rarefaction resulted in the loss of 105 low-abundance OTUs from the 16S data, but no OTUs were lost from the other amplicon data sets. The results from all Aitchison distance-based beta diversity analyses presented in the main manuscript were confirmed using Bray-Curtis, Jaccard, and weighted and unweighted UniFrac distance-based analyses. These four metrics represent the four most-used metrics in ecological

and microbiome-based statistics; the Bray-Curtis and weighted UniFrac distances consider taxon abundances, whereas the Jaccard and unweighted UniFrac distances consider only taxon presence/absence. The two UniFrac distances further account for phylogenetic relationships among taxa [21].

For the random forest models, redundancy analysis (RDA), and co-occurrence network analysis, we used genus-level relative abundances for all genera detected in >4 samples with a mean relative abundance >0.005%. This filtering criteria reduced the likelihood that rare or low-abundance taxa would be used to distinguish populations. In the RDA analysis, we constructed separate models with each of bacteria and protists predicted from each of lichens, algae, and plants. We additionally compared models with and without 'population' as a predictor to determine the extent to which diet composition predicted microbiome composition independent of any population effects. To identify hub taxa in our co-occurrence network analysis, we calculated Z-scores for the degree, betweenness centrality, and closeness centrality of each node. Z-scores were calculated separately for the two separate networks that our analysis produced. Genera were considered hubs if the sum of their three Z scores was greater than or equal to three. This method is similar to that employed by previous studies [22, 23] but allows taxa with unusually high scores in one network to still be considered hubs.

Muribaculaceae genome analysis. Given the abundance of Muribaculaceae spp. in caribou samples, we performed a whole-genome analysis of representative species from the Muribaculaceae genera *Paramuribaculum, Muribaculum,* and *Duncaniella*. We suspected that this analysis might reveal metabolic specialization for alpha- vs. beta-glucan degradation in deep-snow and shallow-snow caribou. We downloaded publicly genomes from *Paramuribaculum intestinale* (GCF_003024815.1), *Muribaculum intestinale* (GCF_001688845.2), *Muribaculum gordoncarteri* (GCF_004801635.1), *Muribaculum caecicola* (GCF_004801635.1), *Duncaniella dubosii* (GCF_004803915.1), *Duncaniella freteri* (GCF_004766125.1), *Duncaniella muris* (GCF_003024805.1), and *Duncaniella muricolitica* (GCF_910574735.1). We also included *Phocaeicola dorei* (GCF_902387545.1) and *Phocaeicola vulgatus* (GCF_020885855.1) due to the overall abundance of *Phocaeicola* in our sample, as well as *Mediterranea massiliensis* (GCF_900128475.1) due to the abundance of *Mediterranea* in shallow-snow caribou. We emphasize that these are publicly available genomes from genera of

interest in our study; however, none of the genomes included in this analysis were obtained from caribou or from our sample.

We used dbCAN3 [24] to annotate all carbohydrate-active enzymes (i.e., CAZymes [25]) in each genome. The dbCAN3 workflow performs annotations using three different tool/database combinations: HMMER [26] against the dbCAN CAZyme database, HMMER against the dbCAN-sub database, and DIAMOND [27]. We retained only those genes that were classified using at least two of these three approaches and then counted the number of genes in each genome that were assigned to each glycoside hydrolase (GH) class in the CAZy database, as these are the enzymes involved in carbohydrate degradation. Genes that were assigned to more than one GH class were counted in both classes, and multiple copies of the same gene were counted each time they occurred. Differences in GH composition between genomes were visualized with a heat map (see **Fig. S16**). We specifically searched for the presence and abundance of GH families involved in the degradation of β-(1,3)-glucans: GH5, GH16, GH17, GH55, GH64, GH81, GH128, and GH158 [28].

REFERENCES

1. Martin M. Cutadapt removes adapter sequences from high-throughput sequencing reads. EMBnetjournal. 2011;17(1):10-2.
2. Callahan BJ, McMurdie PJ, Rosen MJ, Han AW, Johnson AJA, Holmes SP. DADA2: High-resolution sample inference from Illumina amplicon data. Nat Methods. 2016;13(7):581-3.
3. Rognes T, Flouri T, Nichols B, Quince C, Mahé F. VSEARCH: a versatile open source tool for metagenomics. PeerJ. 2016;4:e2584.
4. Schloss PD. Amplicon Sequence Variants Artificially Split Bacterial Genomes into Separate Clusters. mSphere. 2021;6(4):10.1128/msphere.00191-21.
5. Kauserud H. ITS alchemy: On the use of ITS as a DNA marker in fungal ecology. Fungal Ecology. 2023;65:101274.
6. Cole JR, Wang Q, Fish JA, Chai B, McGarrell DM, Sun Y, et al. Ribosomal Database Project: Data and tools for high throughput rRNA analysis. Nucleic Acids Res. 2014;42:633-42.
7. Quast C, Pruesse E, Yilmaz P, Gerken J, Schweer T, Yarza P, et al. The SILVA ribosomal RNA gene database project: Improved data processing and web-based tools. Nucleic Acids Res. 2013;41:D590-D6.
8. Abarenkov K, Zirk A, Piirmann T, Pöhönen R, Ivanov F, Nilsson RH, et al. UNITE general FASTA release for fungi. 16.10.2022 ed: UNITE Community; 2022.
9. Wang Q, Garrity George M, Tiedje James M, Cole James R. Naïve Bayesian Classifier for Rapid Assignment of rRNA Sequences into the New Bacterial Taxonomy. Appl Environ Microbiol. 2007;73(16):5261-7.
10. Katoh K, Misawa K, Kuma Ki, Miyata T. MAFFT: a novel method for rapid multiple sequence alignment based on fast Fourier transform. Nucleic Acids Res. 2002;30(14):3059-66.
11. Sasada R, Weinstein M, Danko D, Wolfe E, Tang S, Jarvis K, et al. Progress Towards Standardizing Metagenomics: Applying Metagenomic Reference Material to Develop Reproducible Microbial Lysis Methods with Minimum Bias. J Biomol Tech. 2020;31(Suppl):S30-1.
12. Callahan BJ, Sankaran K, Fukuyama JA, McMurdie PJ, Holmes SP. Bioconductor Workflow for Microbiome Data Analysis: from raw reads to community analyses. F1000Res. 2016;5:1492.
13. Wright ES. DECIPHER: harnessing local sequence context to improve protein multiple sequence alignment. BMC Bioinformatics. 2015;16(1):322.
14. Schliep KP. phangorn: phylogenetic analysis in R. Bioinformatics. 2011;27(4):592-3.
15. Lücking R, Hodkinson BP, Leavitt SD. The 2016 classification of lichenized fungi in the Ascomycota and Basidiomycota – Approaching one thousand genera. The Bryologist. 2016;119(4):361-416.

16. Nguyen NH, Song Z, Bates ST, Branco S, Tedersoo L, Menke J, et al. FUNGuild: An open annotation tool for parsing fungal community datasets by ecological guild. Fungal Ecology. 2016;20:241-8.

17. Mitchell JK, Garrido-Benavent I, Quijada L, Pfister DH. Sareomycetes: more diverse than meets the eye. IMA Fungus. 2021;12(1):6.

18. Hsieh TC, Ma KH, Chao A. iNEXT: an R package for rarefaction and extrapolation of species diversity (Hill numbers). Methods in Ecology and Evolution. 2016;7(12):1451-6.

19. Gloor GB, Macklaim JM, Pawlowsky-Glahn V, Egozcue JJ. Microbiome datasets are compositional: and this is not optional. Frontiers in Microbiology. 2017;8:2224-.

20. Fernandes A, Macklaim JM, Linn T, Reid G, Gloor GB. ANOVA-like differential expression (ALDEx) analysis for mixed population RNA-Seq. PLoS ONE. 2013;8(6):e67019-e.

21. Lozupone C, Knight R. UniFrac: a New Phylogenetic Method for Comparing Microbial Communities. Appl Environ Microbiol. 2005;71(12):8228-35.

22. Manirajan BA, Maisinger C, Ratering S, Rusch V, Schwiertz A, Cardinale M, et al. Diversity, specificity, co-occurrence and hub taxa of the bacterial–fungal pollen microbiome. FEMS Microbiol Ecol. 2018;94(8).

23. Agler MT, Ruhe J, Kroll S, Morhenn C, Kim S-T, Weigel D, et al. Microbial Hub Taxa Link Host and Abiotic Factors to Plant Microbiome Variation. PLoS Biol. 2016;14(1):e1002352.

24. Zheng J, Ge Q, Yan Y, Zhang X, Huang L, Yin Y. dbCAN3: automated carbohydrate-active enzyme and substrate annotation. Nucleic Acids Res. 2023;51(W1):W115-W21.

25. Cantarel BL, Coutinho PM, Rancurel C, Bernard T, Lombard V, Henrissat B. The Carbohydrate-Active EnZymes database (CAZy): an expert resource for glycogenomics. Nucleic Acids Res. 2009;37(Database):D233-D8.

26. Eddy SR. Profile hidden Markov models. Bioinformatics. 1998;14(9):755-63.

27. Buchfink B, Xie C, Huson DH. Fast and sensitive protein alignment using DIAMOND. Nat Methods. 2015;12(1):59-60.

28. Singh RP, Bhardwaj A. β-glucans: a potential source for maintaining gut microbiota and the immune system. Frontiers in Nutrition. 2023;10:1143682.

SUPPLEMENTARY TABLES

Table S1. PCR primers used for amplicon sequencing reactions.

Table S2: Alpha diversity estimates and statistical comparisons.

Results of Levene's tests, ANOVAs and Tukey's post hoc tests for significant differences in species richness ("Observed"), Shannon diversity ("Shannon"), and Faith's phylogenetic diversity ("PD") in each caribou population (top) and herd (bottom). Richness and diversity were estimated via rarefaction and extrapolation using iNext. Abbreviations: DS, deep-snow; SS, shallow-snow; RP, Revelstoke pen; LARS, Large Animal Research Station; BV, Barkerville; SK, Central Selkirks; Hart, Hart Ranges; NCoo, North Cariboo; NCmb, North Columbia; WG, Wells Gray North.

Table S3: Lichen fungal symbiont genera detected in the LARS reindeer.

Prevalence indicates the number of individuals in which each genus was detected; mean relative abundance is the mean relative abundance of all fungal ITS reads.

Table S4: Statistical results from beta diversity comparisons.

Results of permutational multivariate analyses of variance (PERMANOVAs) and tests for homogeneity of multivariate dispersion among caribou populations and deep-snow caribou herds. Pairwise comparisons are shown for among-population comparisons; however, due to the small sample sizes of individual herds, we did not perform pairwise comparisons for among-herd differences. Test results are shown for five commonly used distance metrics (Bray-Curtis, Jaccard, Aitchison, weighted and unweighted UniFrac). Abbreviations: DS, deep-snow; SS, shallow-snow; RP, Revelstoke pen; LARS, Large Animal Research Station.

Table S5: Random forest model confusion matrices.

Confusion matrices for random forest models predicting the population to which a sample belongs as a function of its amplicon community composition. Out-of-bag error rates are shown for the full model.

Table S6: Lichen fungal genera: differential abundance and Gini scores.

Prevalence ('N') and mean relative abundances (%) of all lichen fungal symbiont genera detected in our study. Genera that were not detected in a population are indicated by '-'. We tested for differential abundance using the 'glm' feature in ALDEx2, and adjusted p-values are shown in the column 'Diff Abund.' Genera that had discriminatory power in random forest models are indicated by the mean decrease in the Gini coefficient ('Gini score'), where higher scores indicate more discriminatory taxa. Abbreviations: DS, deep-snow; SS, shallow-snow; RP, Revelstoke pen; LARS, Large Animal Research Station.

Table S7: Algal genera: differential abundance and Gini scores.

For a description of each column, see the legend to Table S6.

Table S8: Plant genera: differential abundance and Gini scores.

For a description of each column, see the legend to Table S6.

Table S9: Bacterial genera: differential abundance and Gini scores.

For a description of each column, see the legend to Table S6.

Table S10: Protist genera: differential abundance and Gini scores.

For a description of each column, see the legend to Table S6.

Table S11: Fungal genera: differential abundance and Gini scores.

For a description of each column, see the legend to Table S6.

Table S12: Mantel test results.

Results of Spearman correlation-based Mantels tests for significant relationships between different amplicon communities or community subsets. Tests are shown for five commonly used distance metrics.

Table S13: Nodes in all-amplicon correlation network.

Nodes were any lichen, algal, bacterial, or protist genera with a relative abundance >0.5% across all samples (with relative abundances defined within each amplicon pool). In the analysis, one smaller network representing deep-snow caribou was completely unconnected to a larger network representing the other samples; the nodes of that network are indicated as "deep-snow." The "hub score" is the sum of the Z-scores for degree, betweenness centrality, and closeness centrality. All network statistics were calculated using CytoScape v3.10.0.

Table S14: Edges in all-amplicon correlation network.

Edges were defined as strong ($r > 0.6$) and significant (FDR-adjusted $p<0.01$) Spearman correlations between the relative abundances of any two lichen, algal, bacterial, or protist genera.

SEQUENCING OVERVIEW

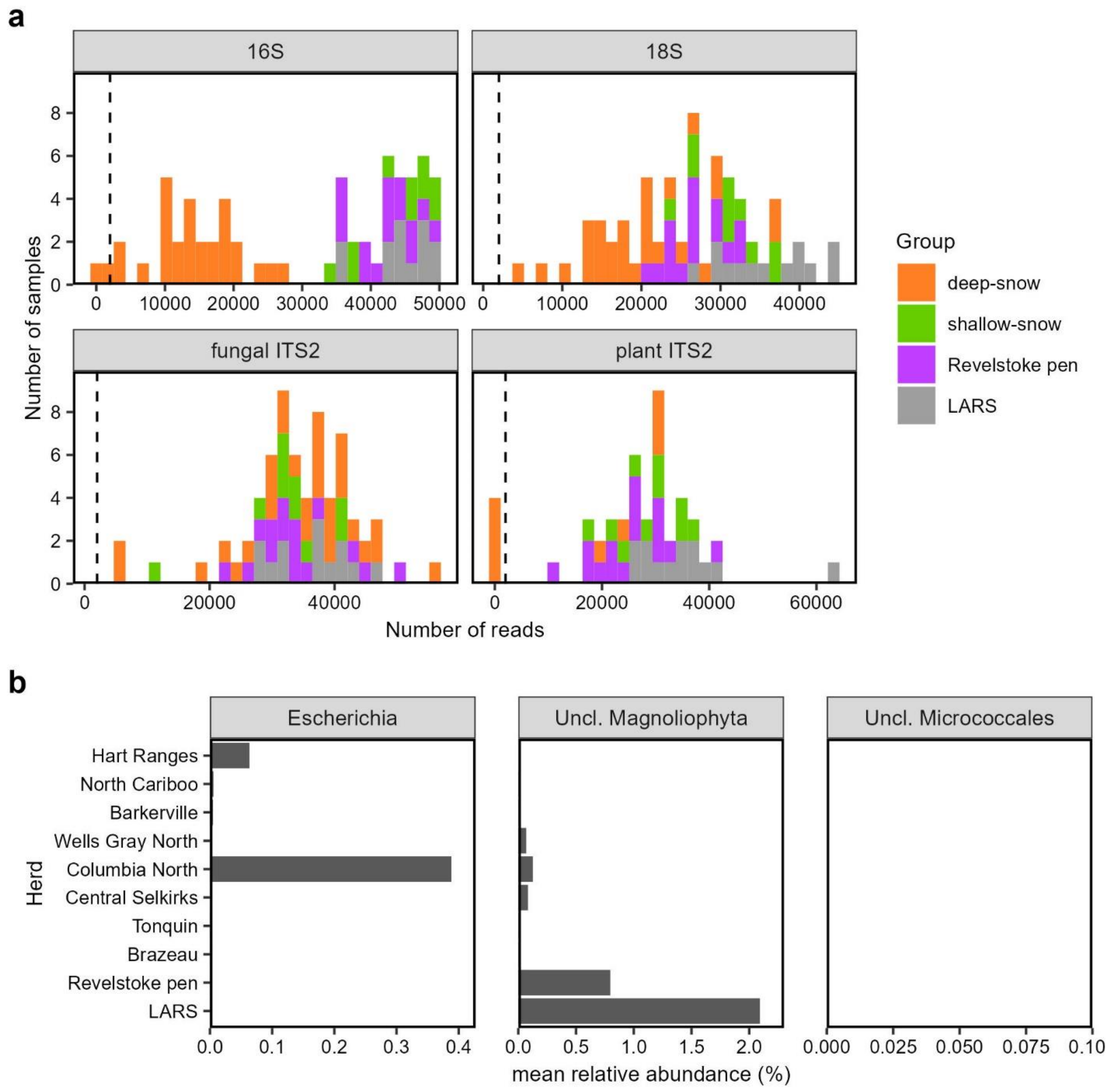

Fig. S1: Sequencing read counts and negative controls.

(**a**) Histogram showing number of reads per sample for each amplicon. Samples are colored by study population, and samples to the left of the vertical black line were removed from analysis because they produced fewer than 2,000 reads. Note that samples with 0 reads for an amplicon are not shown. (**b**) Relative abundances of the three OTUs detected across the two negative control samples. All three OTUs (two 16S amplicons and one 18S amplicon) were removed from downstream analyses.

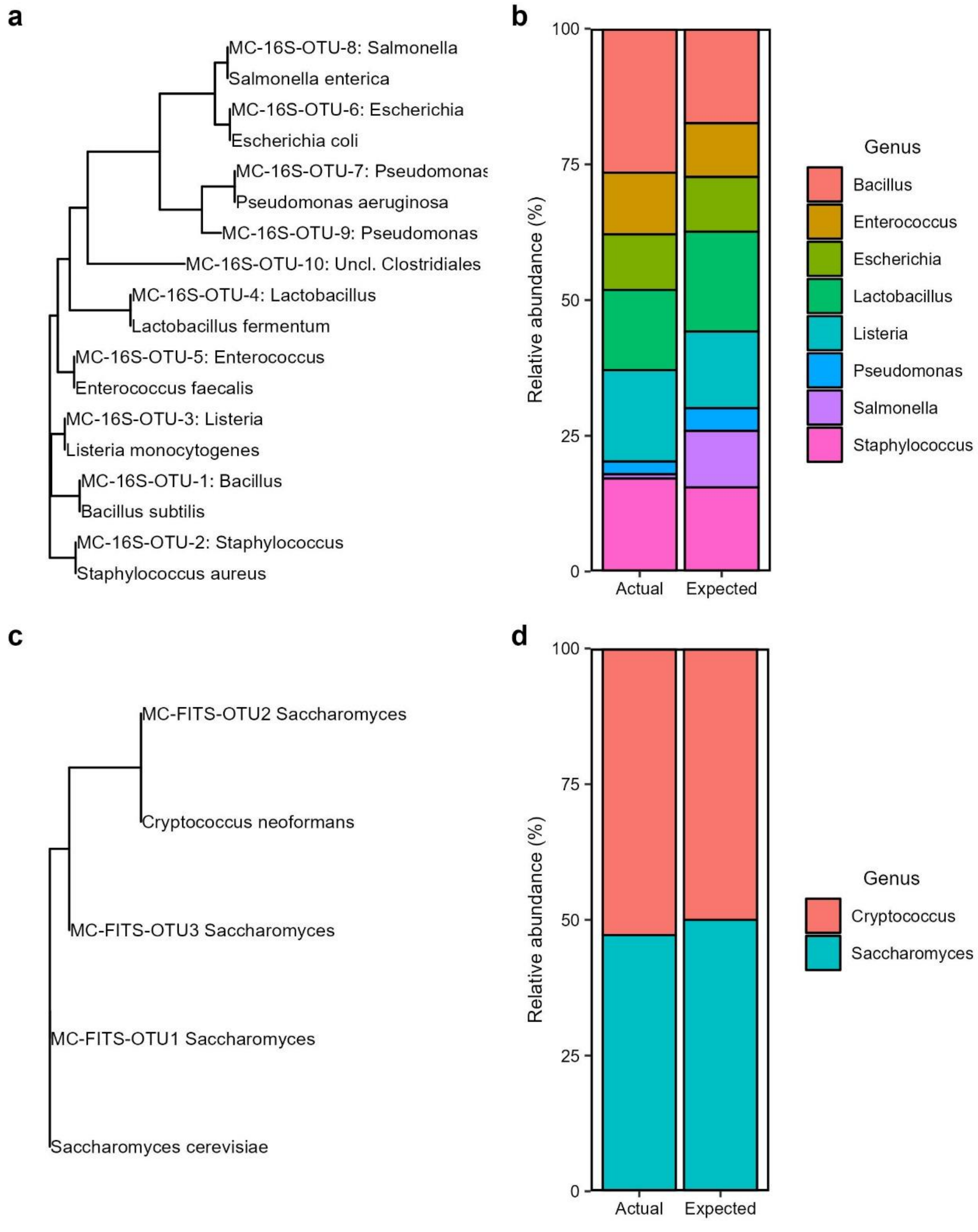

Fig. S2: Mock community sequencing results.

Samples were sequenced alongside the ZymoBIOMICS Microbial Community DNA Standard (ZymoBIOMICS, Irvine, CA). (**a**) Phylogenetic tree for 16S rRNA gene amplicons from the mock community and reference sequences. (**b**) Measured bacterial genus-level relative abundances compared to expected values from the mock community. (**c, d**) Reproductions of panels **a** and **b** for fungal ITS2 amplicons.

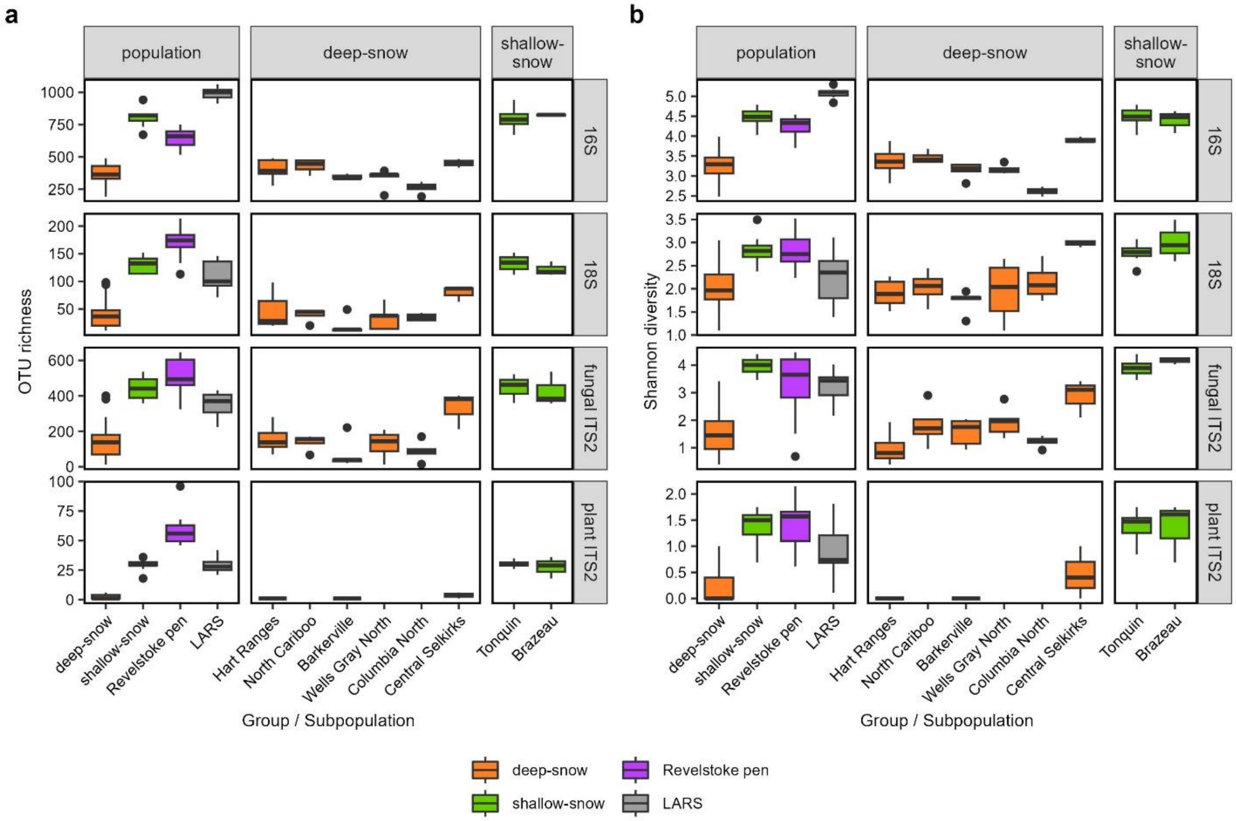

Fig. S3: Alpha diversity overview for the four sequenced amplicons.

(**a**) OTU richness and (**b**) Shannon diversity shown for the four sequenced amplicons (16S rRNA, 18S rRNA, fungal ITS2, and plant ITS2). Values are shown for study populations as well as for individual herds of deep-snow and shallow-snow mountain caribou. See **Table S2** for statistics showing significant differences among populations and herds.

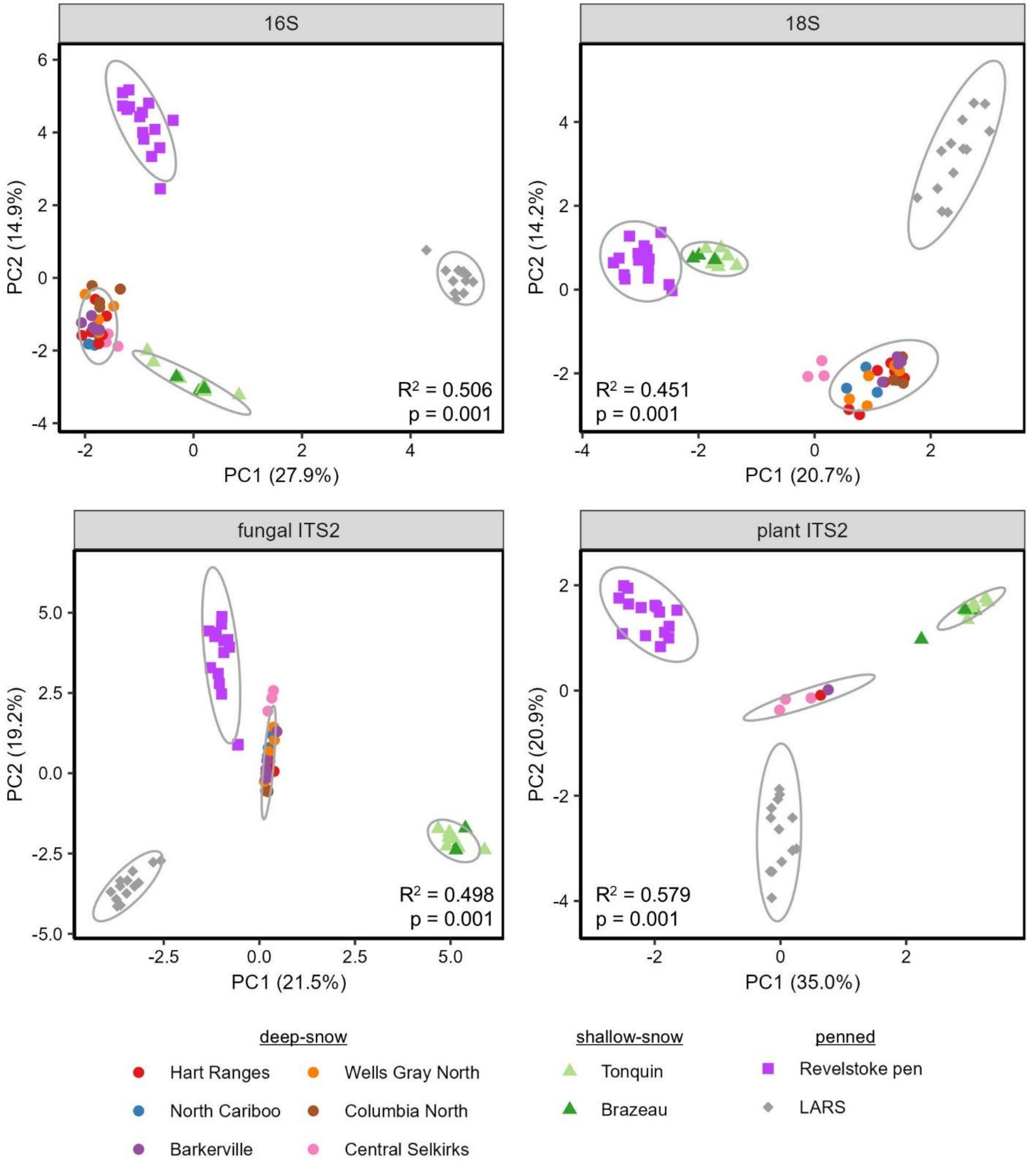

Fig. S4: Beta diversity overview for the four sequenced amplicons.

Aitchison distance-based ordinations of community profiles from the four DNA amplicons sequenced in this study (bacterial 16S rRNA, eukaryotic 18S rRNA, fungal ITS2, and plant ITS2). Values in the bottom right of each panel show the results of a PERMANOVA testing for significant differences among study populations.

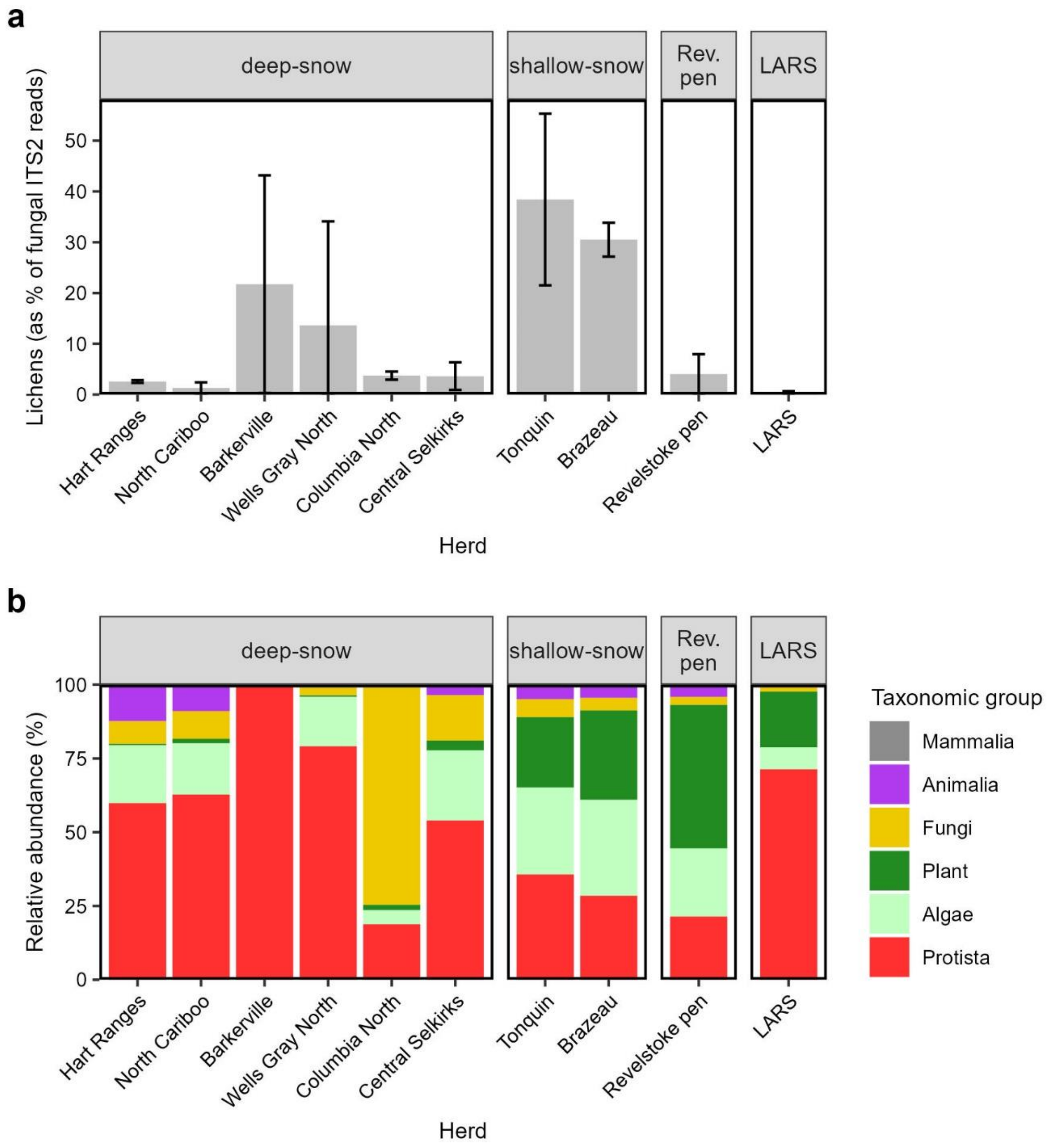

Fig. S5: Amplicon data subsets for the 18S and fungal ITS2 amplicons.

(**a**) Percentage of fungal ITS2 amplicon reads that were assigned to lichen fungal symbionts. Lichen fungal symbionts were identified as any taxa considered “lichenized” by either Lücking et al. [15] or FUNGuild [16]. (**a**) Percentage of 18S rRNA gene amplicon reads that were assigned to each of mammals, other animals, fungi, plants, algae, and protists. Algal and protist reads were each subset for separate analyses; the remaining reads were discarded to avoid overlap with data from the fungal and plant ITS2 amplicons. All bars represent means; error bars show standard deviation.

EVIDENCE OF UNIQUE DIETS

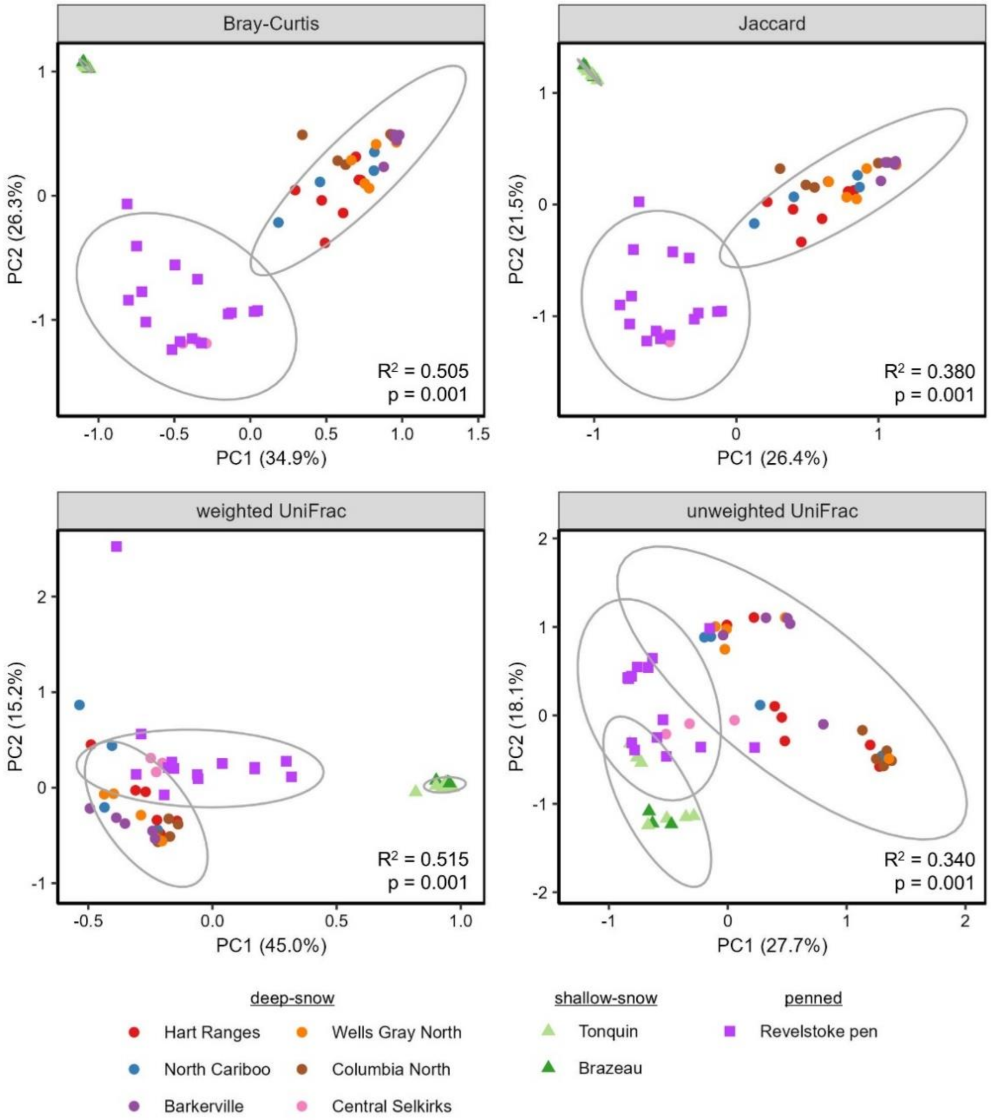

Fig. S6: Alternative distance metrics for lichen fungal symbiont beta diversity. Significant clustering associations separating lichen fungal symbiont communities among study populations, displayed in **Fig. 2b** using the Aitchison distance metric, were preserved when evaluating several common distance metrics used with rarefied data. For the ordinations shown here, read counts were converted to relative abundances prior to principal coordinate analysis based on the Bray-Curtis, Jaccard, and weighted and unweighted UniFrac distances. Note that the LARS population does not appear in these figures because few (<20) fungal ITS2 reads per animal were assigned to lichens. Values in each panel show the results of a PERMANOVA testing for significant differences among study populations.

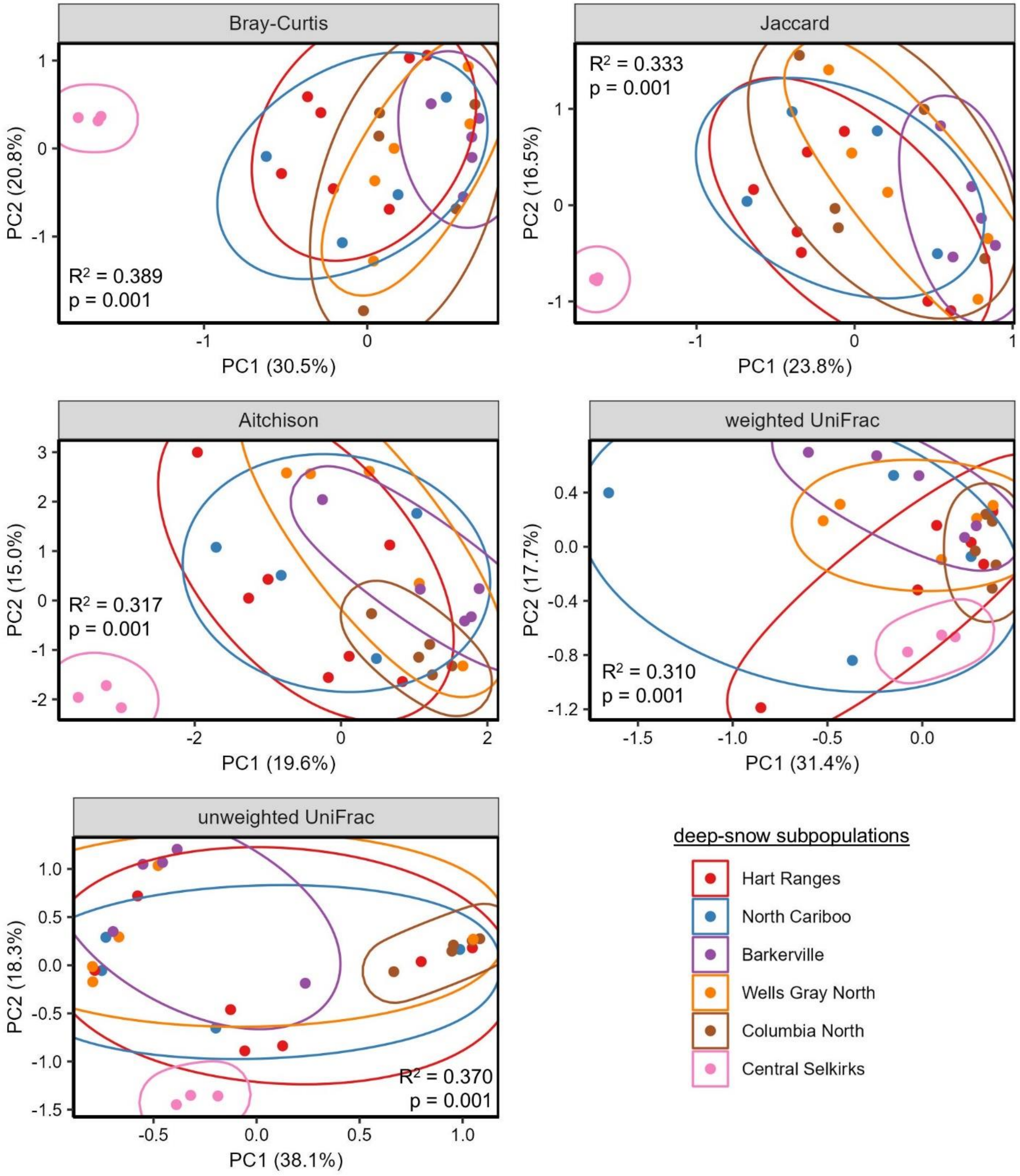

Fig. S7: Among-herd variation in lichen fungal symbiont communities.

Ordinations showing differences in lichen fungal symbiont communities among deep-snow mountain caribou herds, with different panels for different ecological distance metrics. Values in each panel show the results of a PERMANOVA testing for significant differences among study populations.

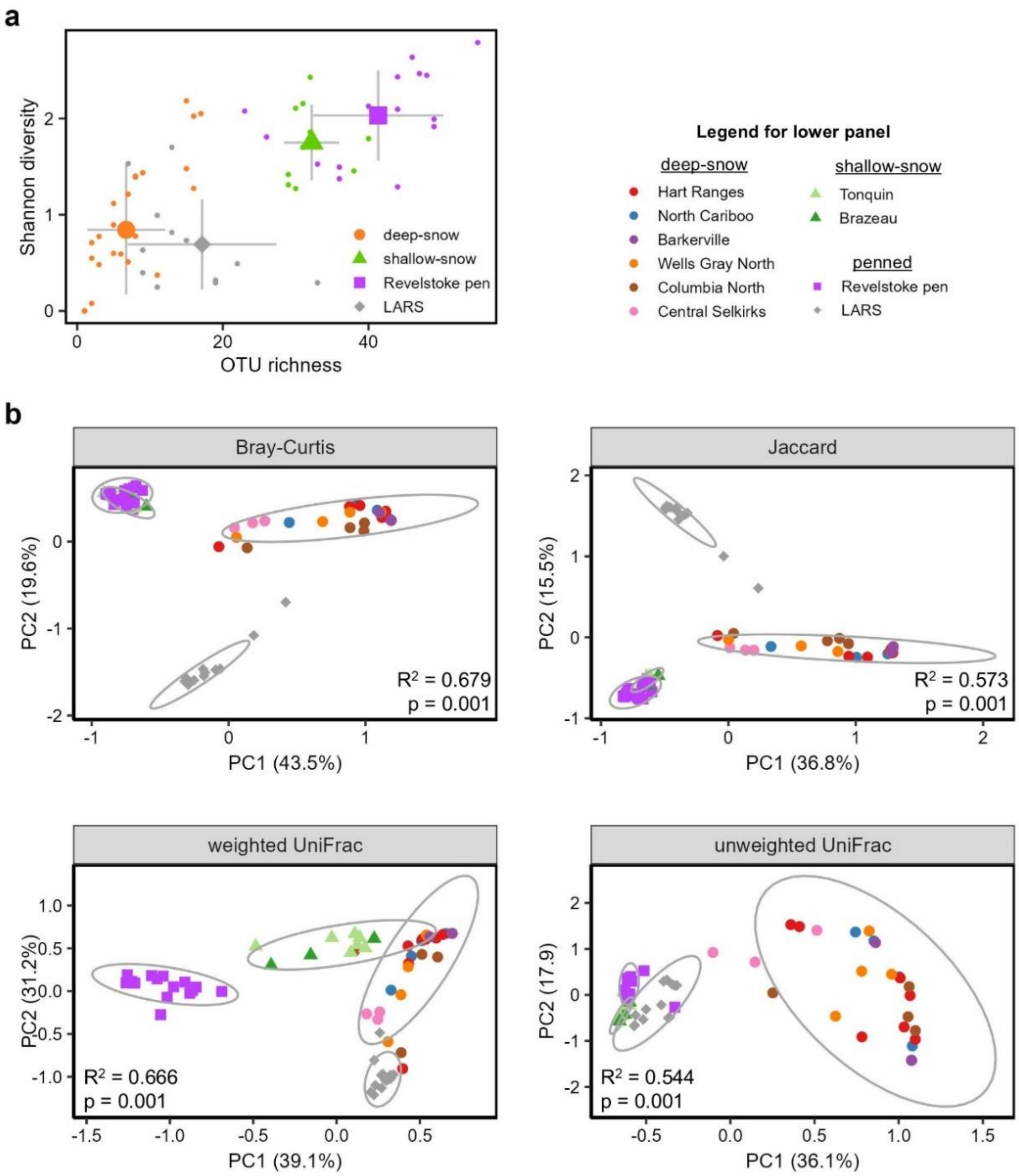

Fig. S8: Algal alpha diversity and alternative distance metrics.

(a) Algal OTU richness and Shannon diversity for all samples. Mean values for each study population are indicated with larger symbols surrounded by an ellipse. Error bars indicate standard deviation. (**b**) Significant clustering associations separating algal communities among study populations, displayed in **Fig. 3a** using the Aitchison distance metric, were preserved when evaluating several common distance metrics used with rarefied data. For the ordinations shown here, read counts were converted to relative abundances prior to principal coordinate analysis based on the Bray-Curtis, Jaccard, and weighted and unweighted UniFrac distances. Values in each panel show the results of a PERMANOVA testing for significant differences among study populations.

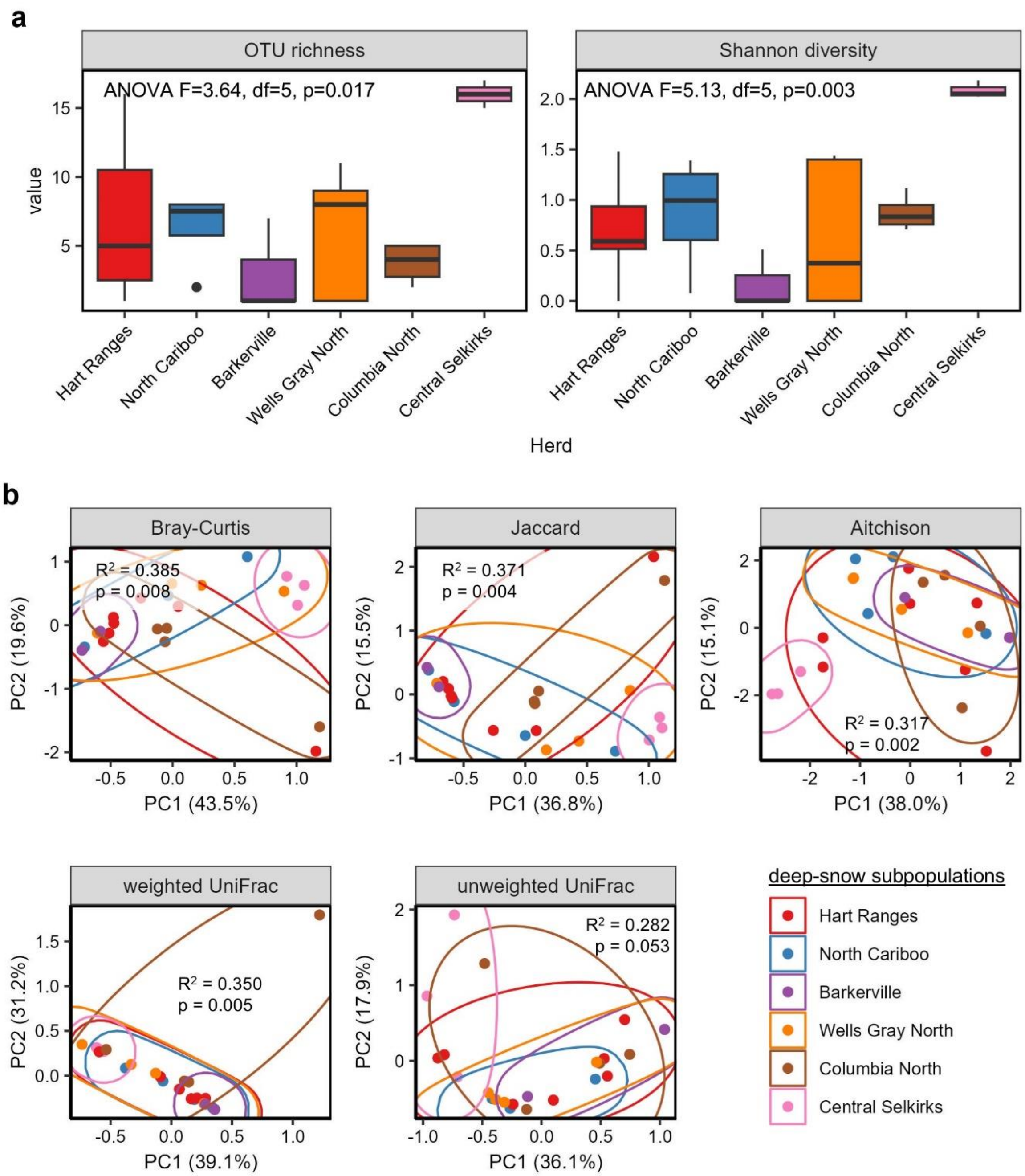

Fig. S9: Among-herd variation in algal communities.

(a) OTU richness (*left*) and Shannon diversity (*right*) for algal communities within deep-snow mountain caribou herds. Given the small sample sizes within each herd, we did not perform pairwise comparisons. **(b)** Ordinations showing differences in algal communities among deep-snow mountain caribou herds, with different panels for different ecological distance metrics. Values in each panel show the results of a PERMANOVA testing for significant differences among study populations.

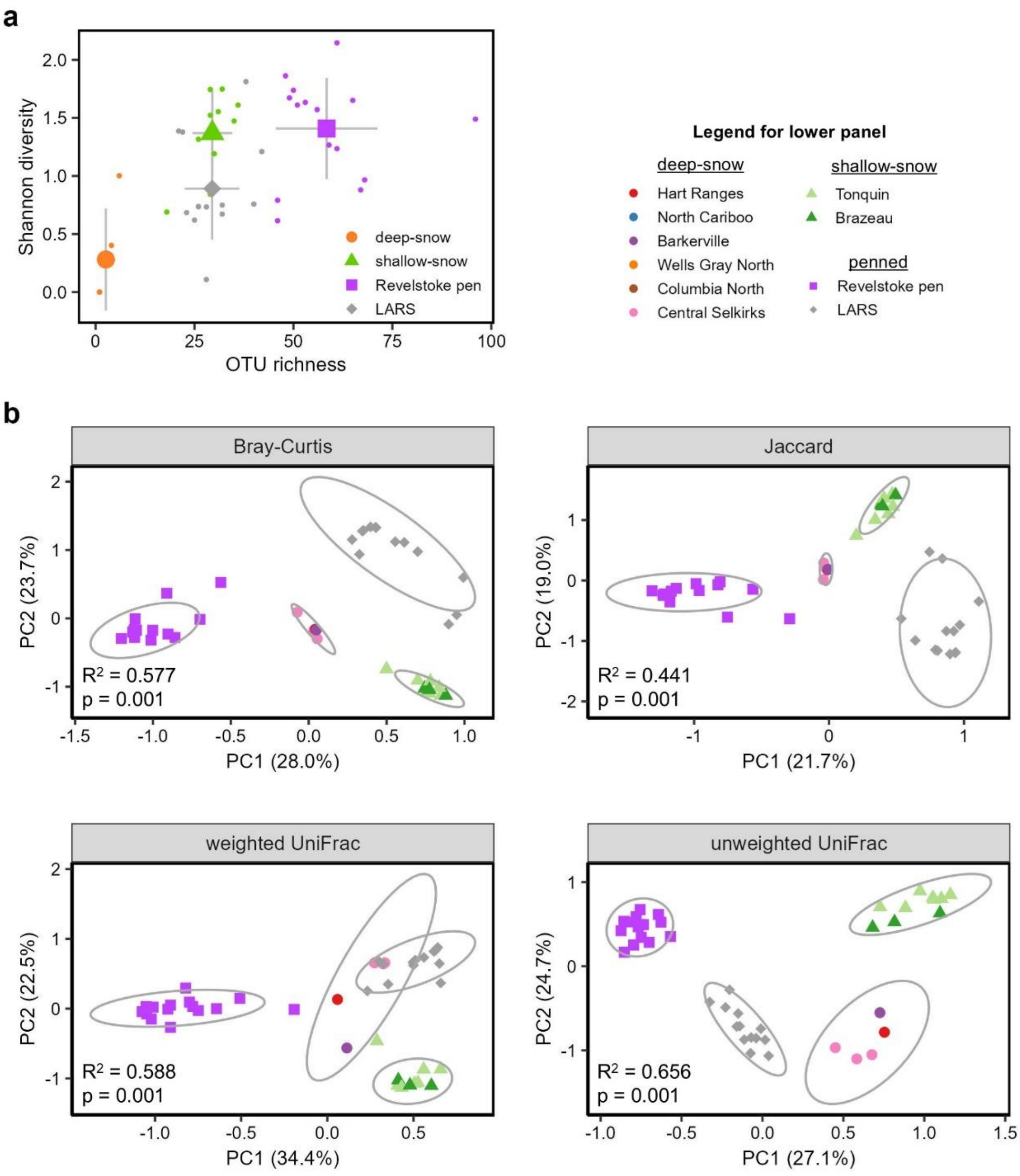

Fig. S10: Plant alpha diversity and alternative distance metrics.

(a) Plant OTU richness and Shannon diversity for all samples. Mean values for each study population are indicated with larger symbols surrounded by an ellipse. Error bars indicate standard deviation. (**b**) Significant clustering associations separating plant communities among study populations, displayed in **Fig. 3c** using the Aitchison distance metric, were preserved when evaluating several common distance metrics used with rarefied data. For the ordinations shown here, read counts were converted to relative abundances prior to principal coordinate analysis based on the Bray-Curtis, Jaccard, and weighted and unweighted UniFrac distances. Values in each panel show the results of a PERMANOVA testing for significant differences among study populations.

EVIDENCE OF UNIQUE MICROBIOME COMPOSITION

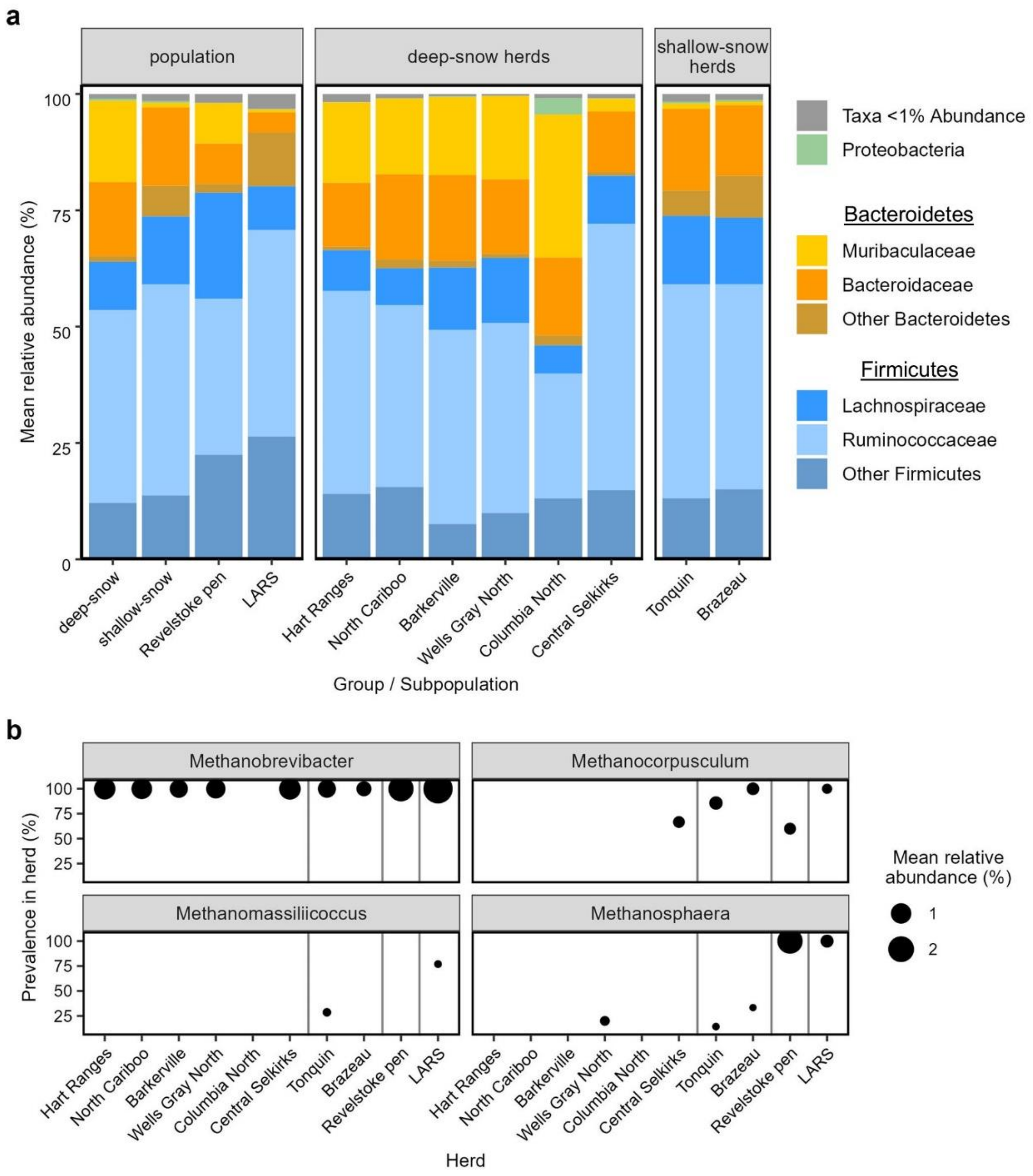

Fig. S11: Bacterial and methanogen relative abundances among herds.

(**a**) Bar charts showing the mean relative abundances of different bacterial taxa in different study populations and herds. (**b**) Methanogens detected in the 16S sequencing data. The prevalence of each methanogen genus, defined as the percentage of samples in each herd that contained sequences from that genus, is shown in the y-axis. The size of each point indicates the mean relative abundance of each methanogen genus among the 16S sequencing reads in each herd.

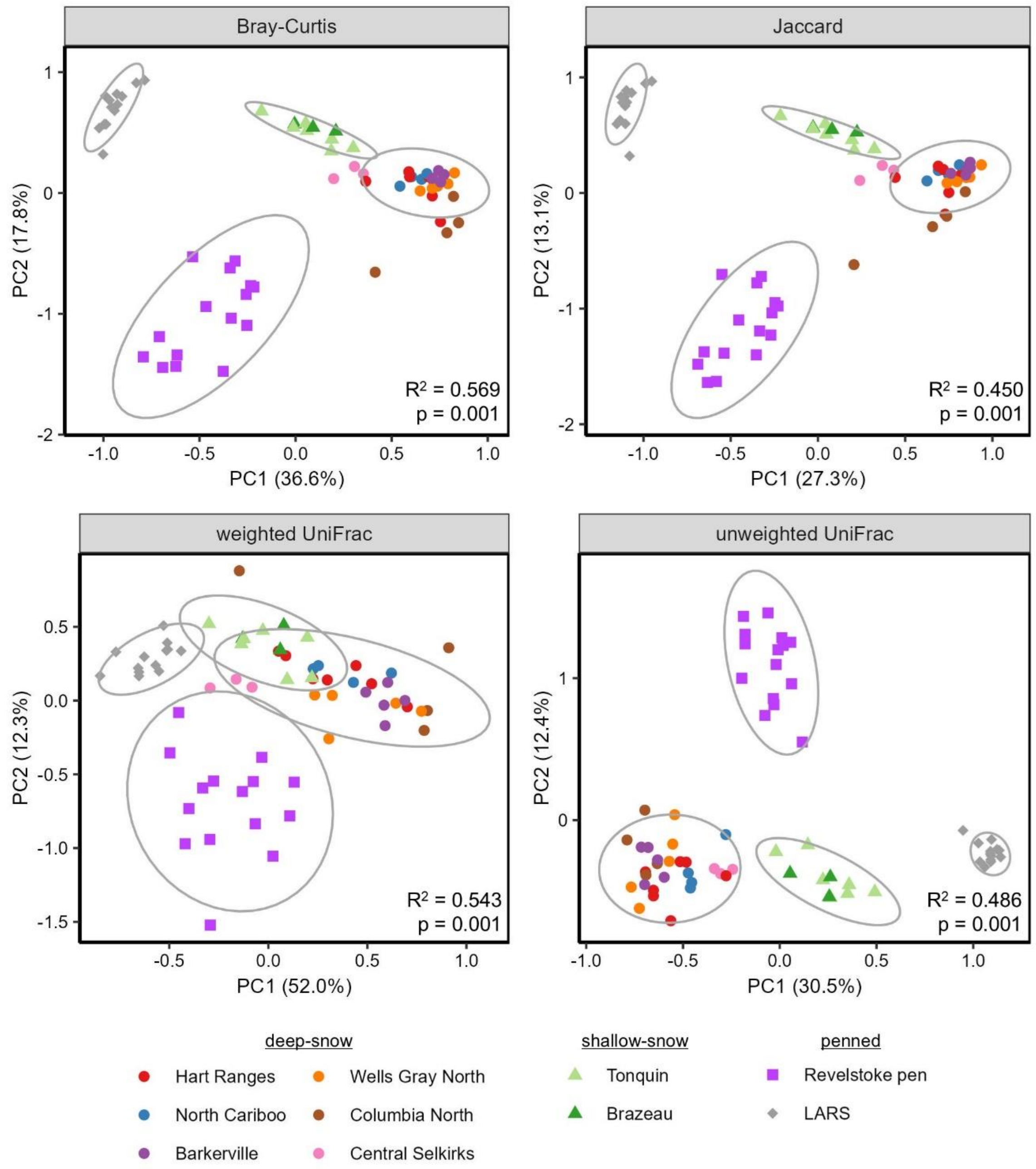

Fig. S12: Alternative distance metrics for bacterial beta diversity.

Significant clustering associations separating bacterial communities among study populations, displayed in **Fig. 4b** using the Aitchison distance metric, were preserved when evaluating several common distance metrics used with rarefied data. For the ordinations shown here, read counts were rarefied prior to principal coordinate analysis based on the Bray-Curtis, Jaccard, and weighted and unweighted UniFrac distances. Values in each panel show the results of a PERMANOVA testing for significant differences among study populations.

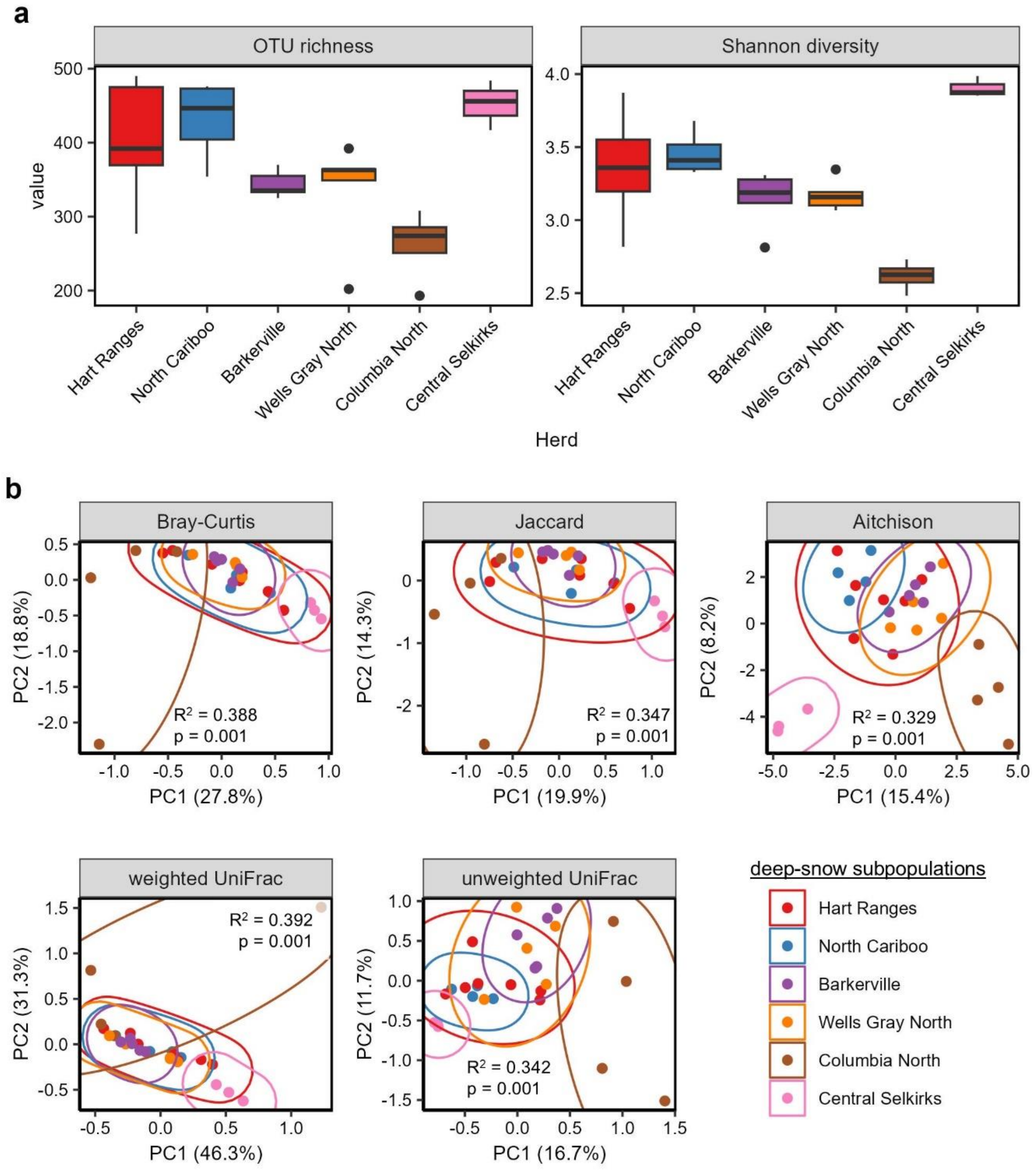

Fig. S13: Among-herd variation in bacterial communities.

(a) OTU richness (*left*) and Shannon diversity (*right*) for bacterial communities within deep-snow mountain caribou herds. Given the small sample sizes within each herd, we did not perform pairwise comparisons. **(b)** Ordinations showing differences in bacterial communities among deep-snow mountain caribou herds, with different panels for different ecological distance metrics. Values in each panel show the results of a PERMANOVA testing for significant differences among study populations.

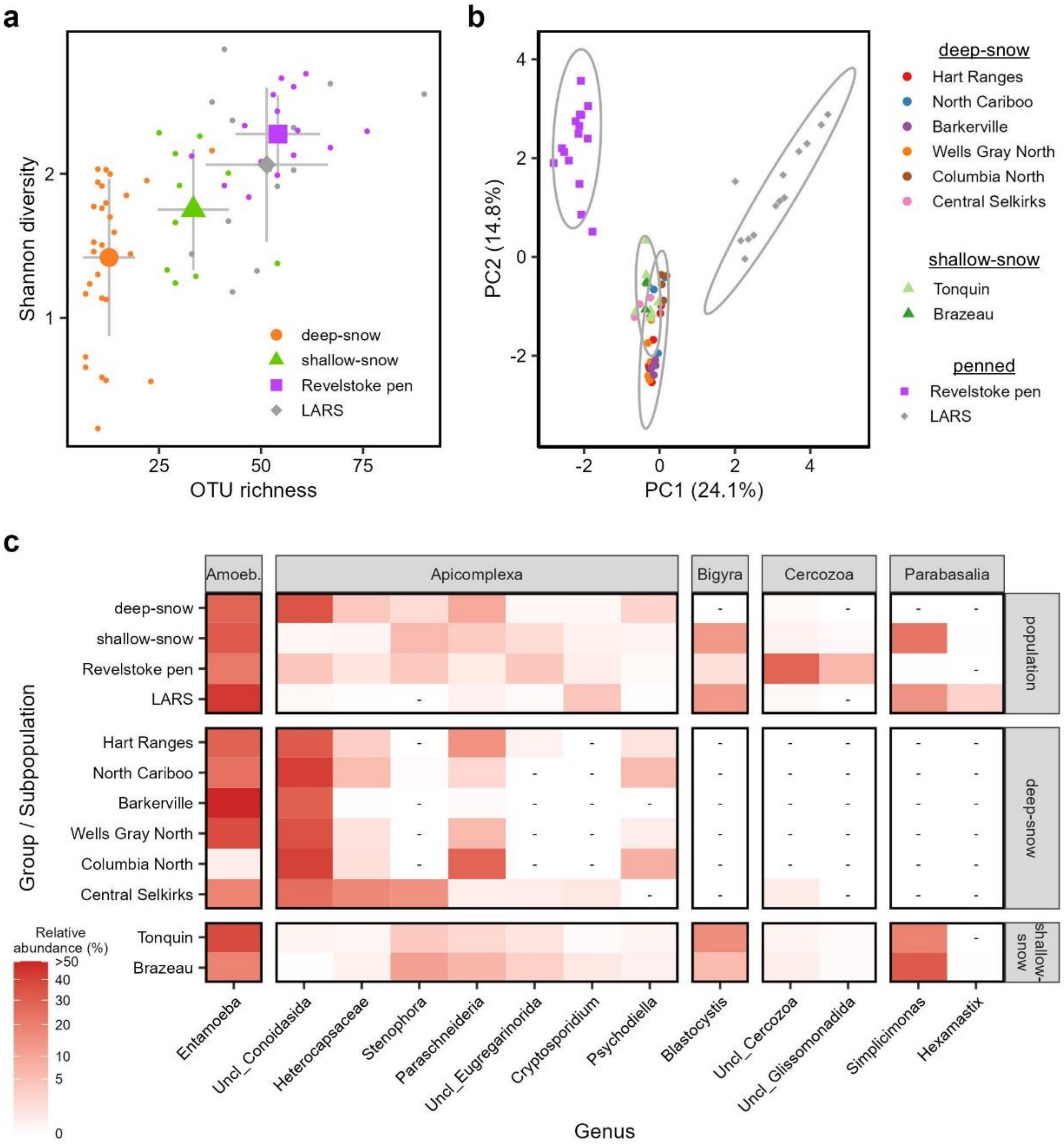

Fig. S14: Protist community diversity and composition.

(a) OTU richness and Shannon diversity are shown for all samples. Mean values for each study population are indicated with larger symbols surrounded by an ellipse. Error bars indicate standard deviation. **(b)** Aitchison distance-based principal coordinate analysis showing differences in protist community composition among study populations. **(c)** Heat map showing significantly differentially abundant protist genera among study populations and herds. The dashes (-) indicate genera that were not detected in a study population. Differentially abundant genera were defined as genera with an FDR-corrected p-value < 0.05 and overall abundance >0.5% across all samples.

OTHER AMPLICON SIGNATURES

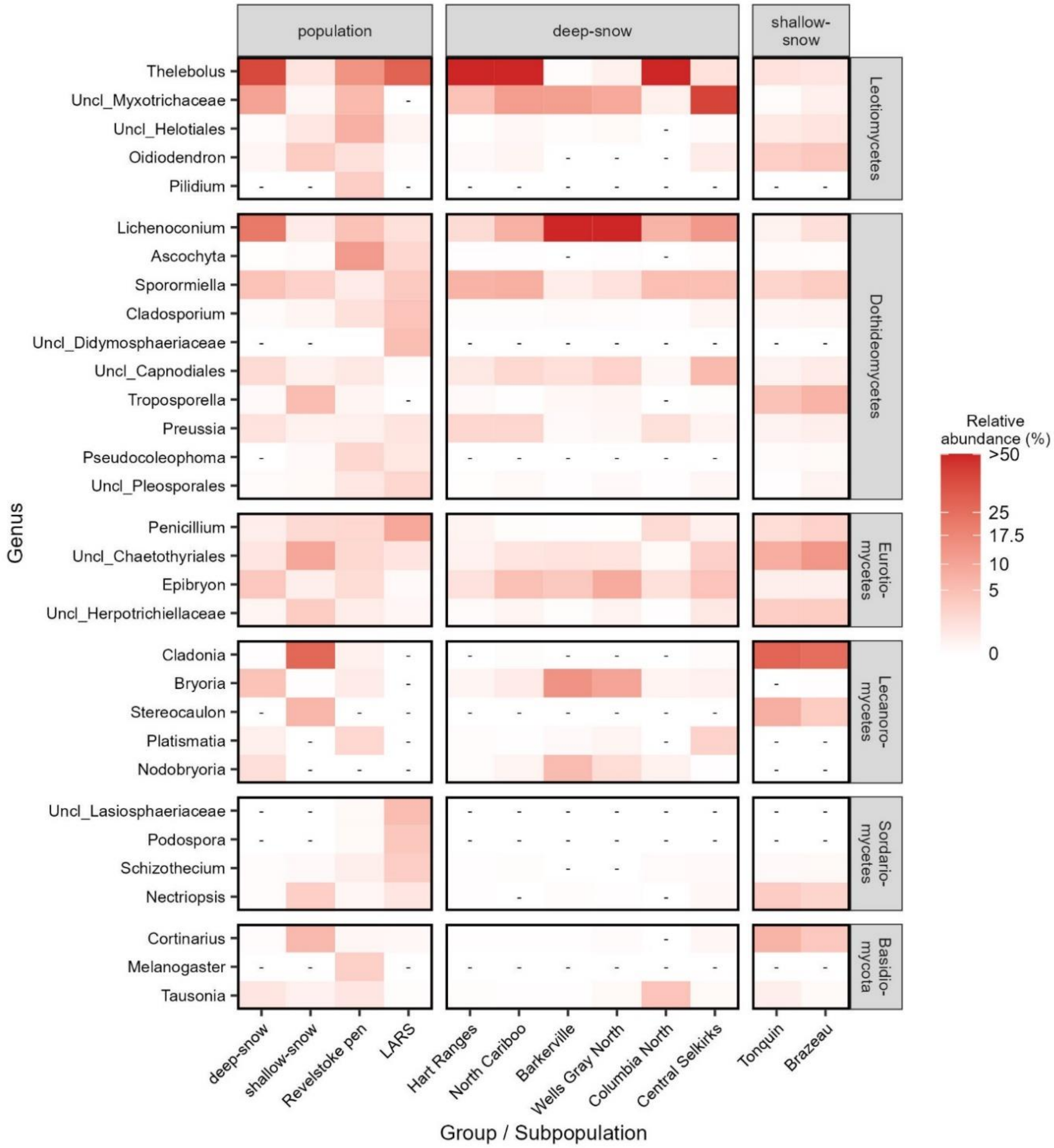

Fig. S15: Relative abundances of fungal genera detected with the fungal ITS2 primers. Heat map showing significantly differentially abundant fungal genera among study populations and herds. Unlike **Fig. 2**, which shows only lichen fungal symbionts, this chart shows the relative abundances of all differentially abundant fungal taxa as a proportion of all fungal ITS2 sequences in each population or herd. Dashes (-) indicate genera that were not detected in a study population. Differentially abundant genera were defined as genera with an FDR-corrected p-value < 0.05 and overall abundance >0.5% across all samples.

Muribaculaceae genome analysis

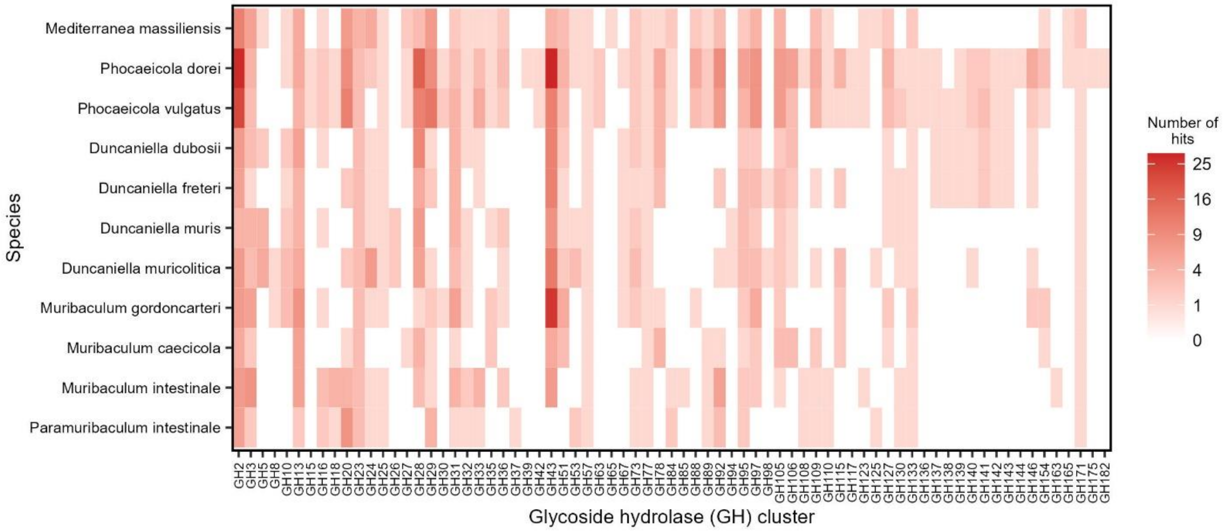

Fig. S16: Glycoside hydrolase content of representative Muribaculaceae, *Phocaecicola*, and *Mediterranea* genomes.

We downloaded publicly available genomes from representative species in the genera *Paramuribaculum*, *Muribaculum*, *Duncaniella*, *Phocaecicola*, and *Mediterannea* to determine whether among-populations differences in microbiome composition may be driven by the functional activity of these significantly differentially abundant taxa, and especially by the enzymatic machinery for digesting β-glucans (see **Supplementary Methods**). Genomes were annotated against the CAZy database [25] using dbCAN3 [24]. The heat map shows the number of genes assigned to each glycoside hydrolase (GH) cluster; note the square root transform. The GH clusters we detected that are involved in β-glucan degradation are GH5 and GH16; other GH clusters involved in β-glucan degradation but not detected in these genomes are GH17, GH55, GH64, GH81, GH128, and GH158 [28]. Nevertheless, we emphasize that the available genomes do not come from species found in the caribou gut, and microbial functional potential varies widely even among species or strains from the same genus. It therefore remains possible that microbiome variation may still be due to the enzymatic machinery relevant to digestion or animal health, whether that be the polysaccharide composition of fungal cell walls or other factors not considered here.
